# Tellurium nanoparticles as antimicrobial agents for multi-drug-resistant infections

**DOI:** 10.1039/d5ra02635k

**Published:** 2025-09-30

**Authors:** Abiola Samuel Ajayi, Pelumi Adanigbo, Joshua Tunde Olaifa, Miracle Oluwabukunmi Obaleye, Emmanuella Amara Ofoka, Sunday Onyebuchi Ukanwa, Prince Duru, Fagbolade Moshood, Aireguamen I. Aigbodion, Ikhazugbe Hilary Ifijen

**Affiliations:** a Texas Southern University 31000 Cleburne St Houston TX 77004 USA; b Department of Chemistry and Biochemistry, George Mason University Fairfax VA USA; c Department of Crop Science, University of Ilesa Ilesa Osun State Nigeria; d Department of Veterinary Physiology and Pharmacology, Texas A&M University College Station Texas USA; e Emergency Medicine Department, University of Tennessee Medical Center 1924 Alcoa Hwy Knoxville TN 37920 USA; f Department of Biological Sciences 219 Harned Hall Mississippi State MS 39762 USA; g Department of Physical Chemistry, Benson Idahosa University Benin City Edo State Nigeria; h Department of Research Outreach, Rubber Research Institute of Nigeria Nigeria larylans4u@yahoo.com; i Department of Bioengineering, Cyprus International University 99258 Nicosia North Cyprus Turkey; j Department of Microbiology, University of Ilesa Ilesa Osun State Nigeria

## Abstract

The rise of multi-drug-resistant (MDR) infections has driven interest in alternative antimicrobial agents, with tellurium nanoparticles (TeNPs) emerging as a promising solution. TeNPs possess unique physicochemical properties, including controlled size, shape, and surface chemistry, which contribute to their potent antimicrobial activity. Their mechanisms of action include reactive oxygen species (ROS) generation, membrane disruption, inhibition of essential microbial proteins and enzymes, and direct damage to DNA and RNA. These multifaceted interactions enable TeNPs to combat a broad spectrum of bacterial and fungal pathogens effectively. In addition to their direct antimicrobial effects, TeNPs have demonstrated efficacy in disrupting and preventing biofilm formation, a key factor in persistent infections. Their application in treating infected wounds has shown promise by reducing microbial burden while promoting wound healing. Notably, TeNPs exhibit synergistic effects when combined with conventional antibiotics, enhancing bacterial eradication and potentially mitigating resistance development. However, concerns remain regarding their cytotoxicity, biodegradability, and long-term clearance in mammalian systems. Addressing these issues through surface modifications and controlled release strategies is essential for safe biomedical applications. Despite their potential, challenges such as scalable production, stability, and regulatory approval hinder widespread use. Future research will focus on advanced functionalization to enhance selectivity, emerging applications such as biofilm disruption and antiviral therapies, and integration with smart technologies for infection monitoring. A structured roadmap for clinical translation is necessary to move TeNP-based therapies from experimental studies to medical practice. The continued development of TeNPs could revolutionize antimicrobial strategies and address the global antibiotic resistance crisis.

## Abstract 2

Antibiotic resistance limits conventional drugs, but tellurium nanoparticles (TeNPs) offer a promising alternative, effectively targeting resistant microbes and providing a novel strategy against multi-drug-resistant infections.

## Graphical abstract

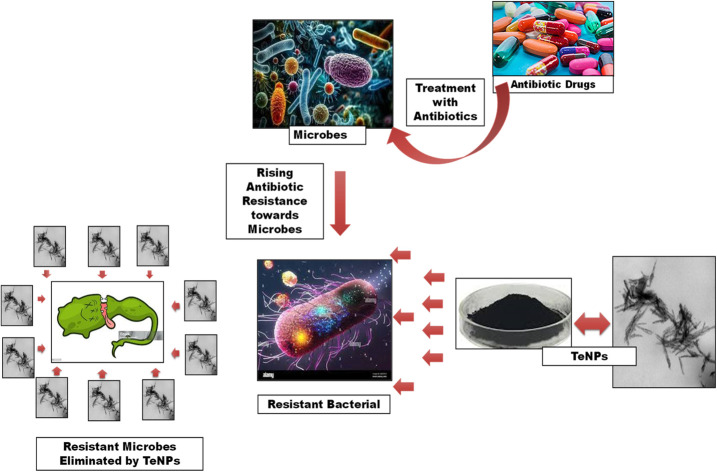

## 1 Introduction

Multi-drug-resistant (MDR) infections are a growing global health threat, significantly impacting public health by prolonging hospital stays, increasing healthcare costs, and raising mortality rates, particularly among vulnerable populations such as the immunocompromised and elderly.^1–5^ The World Health Organization (WHO) has recognized antimicrobial resistance (AMR) as one of the top global health challenges, driven by factors such as antibiotic overuse, poor infection control, and a lack of new drug development.^6–10^ The economic burden of AMR is substantial, with billions of dollars spent annually on treating resistant infections.^11–13^

In response to the limitations of traditional antibiotics, nanotechnology has emerged as a promising solution. Nanoparticles (NPs) offer significant advantages over conventional antimicrobial agents due to their small size, high surface area, and unique physicochemical properties, enabling efficient microbial cell penetration and the release of reactive species to damage microbial membranes, proteins, and DNA.^14,15^ Among various nanoparticles, tellurium-based nanoparticles (TeNPs) stand out due to their ability to generate reactive oxygen species (ROS) under specific conditions, making them effective against a broad range of MDR pathogens. TeNPs also exhibit excellent biocompatibility and tunable surface properties, which enhance their antimicrobial efficacy while minimizing human cell toxicity.^16–21^

This review explores the potential of TeNPs as next-generation antimicrobial agents, focusing on their mechanisms of action, applications in combating MDR infections, and the challenges and opportunities associated with their clinical use.

## 2 Properties of TeNPs

TeNPs possess a unique combination of physicochemical properties that make them particularly suitable for addressing pressing biomedical challenges, including the treatment of multi-drug-resistant infections. These properties are largely determined by their structural, electronic, and optical characteristics, which are further influenced by their morphology, size, and surface modifications.^22,23^

One of the defining features of TeNPs is their structural versatility, as they can be synthesized in a variety of morphologies such as nanowires, nanotubes, nanorods, and spherical nanoparticles. These different shapes contribute significantly to their functionality. For instance, one-dimensional nanowires and nanotubes exhibit a high aspect ratio, resulting in an increased surface area-to-volume ratio. This enhanced surface area enables more effective interaction with microbial cells, facilitating greater antimicrobial activity.^24,25^ Additionally, their elongated shape allows for deep penetration into microbial membranes, disrupting their integrity and function. In contrast, spherical TeNPs are more uniform in dispersibility and isotropic in nature, offering enhanced colloidal stability and ease of functionalization for targeted applications. These morphological variations allow TeNPs to be tailored for specific biomedical purposes, depending on the intended mode of action.^25^

TeNPs are also characterized by their remarkable electronic properties, which stem from tellurium's intrinsic helical chain structure in its crystalline phase. As a p-type semiconductor, tellurium exhibits anisotropic electrical conductivity, enabling it to interact with microbial cells in unique ways. TeNPs can generate reactive oxygen species (ROS) under specific conditions, such as light irradiation or the presence of reducing agents in biological systems.^26,27^ This ROS generation capability plays a central role in their antimicrobial activity, as it leads to oxidative stress within microbial cells, ultimately damaging essential biomolecules like lipids, proteins, and nucleic acids. Furthermore, the high redox potential of TeNPs disrupts microbial metabolic pathways, contributing to their efficacy against a broad range of pathogens, including drug-resistant strains.^28^

The optical properties of TeNPs further enhance their utility in antimicrobial applications. These nanoparticles exhibit strong absorption in the visible to near-infrared (NIR) region, a characteristic that enables their use in photothermal therapy. Upon exposure to light, TeNPs can convert absorbed energy into heat, which can effectively damage microbial cells. Additionally, their photocatalytic activity is another critical attribute.^29^ When excited by light, TeNPs produce ROS, which amplify their ability to target and destroy microbial membranes, proteins, and DNA. The optical properties of TeNPs are also highly shape-dependent, with nanowires and nanotubes displaying unique anisotropic behaviour, while spherical nanoparticles exhibit isotropic optical responses. This versatility in optical behaviour enables TeNPs to be applied across a range of therapeutic and diagnostic modalities.^30^

Another critical aspect of TeNPs is their high surface area-to-volume ratio, which enhances their reactivity and interaction with microbial cells. This high surface area not only facilitates adsorption of biological molecules, such as proteins and enzymes, but also provides ample sites for surface functionalization. By modifying the surface of TeNPs with polymers, ligands, or other biomolecules, their biocompatibility, targeting specificity, and therapeutic efficacy can be significantly improved. Surface modifications also allow for controlled drug release, enabling the delivery of antimicrobial agents directly to the site of infection while minimizing off-target effects.^25–28^

TeNPs are further distinguished by their excellent stability under a variety of environmental and physiological conditions. This stability ensures that their antimicrobial activity is retained even in complex biological systems. Chemical and thermal stability can be further enhanced through surface engineering, which prevents aggregation and ensures consistent performance. These properties make TeNPs suitable for long-term storage, transport, and use in clinical applications, where consistent efficacy is critical.^28–30^

Generally, the physicochemical properties of TeNPs, including their structural flexibility, electronic capabilities, optical behaviour, high surface area, and stability, render them highly effective and versatile antimicrobial agents. Their ability to disrupt microbial systems through multiple mechanisms, combined with their potential for functionalization and controlled therapeutic delivery, underscores their transformative potential in addressing the global threat of multi-drug-resistant infections. By leveraging these unique properties, TeNPs hold significant promise in advancing next-generation antimicrobial therapies and improving global healthcare outcomes.

## 3 Mechanisms of antimicrobial action

### 3.1 Reactive oxygen species (ROS) generation

TeNPs are well known for their ability to generate reactive oxygen species (ROS), a key factor in their antimicrobial properties. ROS are highly reactive molecules that include free radicals such as superoxide anions (O_2_˙^−^), hydroxyl radicals (˙OH), and non-radical molecules like hydrogen peroxide (H_2_O_2_). These species are produced as a result of the interaction between TeNPs and microbial cells, and their generation plays a significant role in inducing oxidative stress, which leads to cellular damage and eventual microbial cell death.^31,32^

The process of ROS generation in the presence of TeNPs begins when the nanoparticles interact with biological systems, typically microbial cell membranes or other intracellular components. TeNPs have unique electronic properties that make them capable of catalyzing redox reactions. The surface of TeNPs can undergo electron transfer processes, which are triggered by exposure to external stimuli such as light, heat, or even the physiological conditions of the environment. For instance, when TeNPs come into contact with electron-donating species like water or oxygen, they can transfer electrons to molecular oxygen (O_2_), resulting in the formation of superoxide anions (O_2_˙^−^).^32^

Once superoxide radicals are produced, they can further interact with other molecules, leading to the generation of secondary ROS such as hydrogen peroxide (H_2_O_2_) and hydroxyl radicals (˙OH). This cascade of ROS formation is often referred to as the “oxidative burst,” where each type of ROS has a distinct role in attacking various cellular components. The ROS generated by TeNPs are highly reactive and capable of damaging multiple cellular structures, starting with the microbial cell membrane.^33^ The lipid bilayer of the membrane is particularly vulnerable to ROS, which can induce lipid peroxidation. This process leads to the formation of toxic byproducts that disrupt the integrity of the membrane, causing an increase in membrane permeability. As a result, essential intracellular contents such as ions, proteins, and nucleic acids leak out, disrupting the cell's homeostasis and leading to cell death.^34^

In addition to membrane damage, ROS can also attack intracellular macromolecules such as proteins, lipids, and DNA. The oxidation of proteins by ROS can lead to denaturation, inactivation, or aggregation, affecting cellular functions and enzymatic processes. ROS can also cause oxidative damage to DNA, including strand breaks, base modifications, and cross-linking, which impairs replication and transcription, ultimately leading to cell death or mutations. In bacteria, this oxidative stress can overwhelm the cell's repair mechanisms, especially when antioxidant defenses are insufficient or overwhelmed by the continuous ROS generation from TeNPs.^34,35^

Furthermore, the ability of TeNPs to generate ROS can also affect other cellular processes, such as energy production and cell signalling. Mitochondrial damage due to oxidative stress can impair ATP production, further compromising cellular functions. In some cases, the accumulation of ROS can activate signalling pathways associated with apoptosis or necrosis, leading to the programmed death of microbial cells. One of the key advantages of ROS generation by TeNPs is that it provides a mechanism of action that is not easily bypassed by microbial resistance mechanisms.^36^ Unlike conventional antibiotics, which target specific molecular pathways that bacteria can mutate or modify to develop resistance, the oxidative stress induced by ROS is a more generalized mechanism of action. This makes ROS-producing TeNPs particularly effective against multi-drug-resistant (MDR) strains, as the pathogens are less likely to evolve mechanisms to neutralize or counteract oxidative damage.^37^

The generation of ROS by TeNPs plays a crucial role in their antimicrobial activity. By catalyzing the production of superoxide anions (O_2_˙^−^), hydrogen peroxide (H_2_O_2_), and hydroxyl radicals (˙OH), TeNPs initiate oxidative stress within microbial cells, leading to damage of the cell membrane, proteins, and DNA. This cascade of oxidative damage overwhelms the microbial cell's defence mechanisms, ultimately resulting in cell death. The ability of TeNPs to produce ROS provides an effective and versatile mechanism for combating a wide range of pathogens, including those that are resistant to conventional antimicrobial agents.^37–39^

**Fig. 1:**
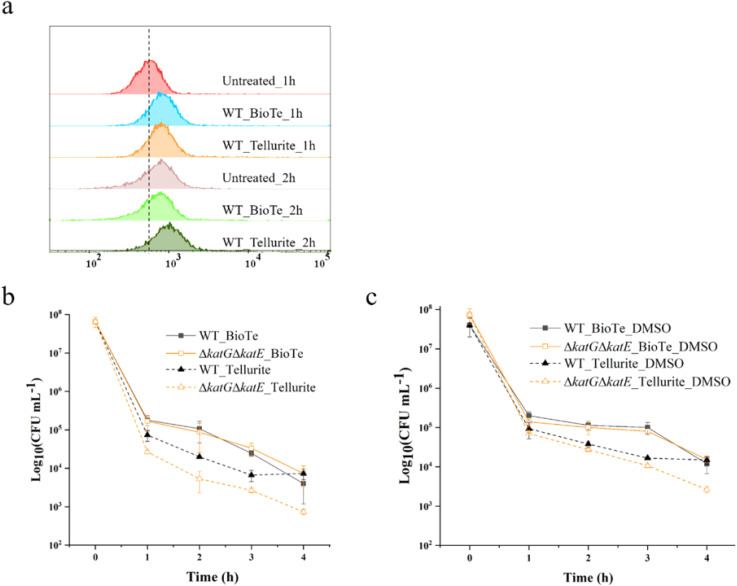
Reactive Oxygen Species (ROS) generation and its role in BioTe- and tellurite-induced cell death. (a) ROS levels in cells treated with BioTe or tellurite. (b) Sensitivity of the ΔkatEΔkatG mutant to the bactericidal effects of BioTe and tellurite. (c) Influence of DMSO on the antimicrobial activity of BioTe and tellurite. Both compounds were used at a concentration of 3× MIC. Data represent the mean ± SD (*n* = 3).^40^

Tang *et al.* (2022) investigated the role of reactive oxygen species (ROS) in the antibacterial mechanism of rod-shaped biologically synthesized tellurium nanoparticles (BioTe) and compared their effects with tellurite.^40^ Their study demonstrated that while ROS production is often implicated in the antimicrobial activity of various nanoparticles, its role in BioTe-mediated bacterial killing is limited. The researchers measured intracellular ROS levels in *Escherichia coli* following exposure to BioTe or tellurite at three times the minimum inhibitory concentration (MIC). After one hour of treatment, both BioTe- and tellurite-exposed cells exhibited an increase in ROS levels compared to untreated controls, confirming that both agents initially induced oxidative stress (Fig. 1a). However, after two hours of treatment, the ROS levels in BioTe-treated cells returned to baseline, while tellurite-treated cells maintained elevated ROS levels. This finding suggests that although BioTe triggers a transient ROS surge, it does not sustain oxidative stress at levels that exceed *E. coli*'s defensive threshold, distinguishing it from tellurite.

The study further examined the role of specific ROS detoxification mechanisms in *E. coli* by assessing the survival of mutant strains lacking key antioxidant enzymes. Wild-type *E. coli* relies on superoxide dismutases (SodA, SodB, and SodC) to convert superoxide (O_2_˙^−^) into hydrogen peroxide (H_2_O_2_), which is subsequently decomposed into water and oxygen by catalases (KatG and KatE). The ΔkatGΔkatE double mutant, which lacks both catalases, displayed heightened sensitivity to tellurite exposure, supporting the idea that ROS accumulation plays a critical role in tellurite-induced bacterial toxicity (Fig. 1b). However, this mutant showed no increased susceptibility to BioTe, further reinforcing the conclusion that ROS production is not a primary contributor to BioTe's antibacterial effect. Additionally, the hydroxyl radical scavenger dimethyl sulfoxide (DMSO) provided significant protection against tellurite toxicity but had no effect on BioTe-treated cells (Fig. 1c). Since hydroxyl radicals (˙OH) are the most reactive and damaging ROS species, their neutralization typically mitigates ROS-mediated bacterial killing. The ineffectiveness of DMSO in counteracting BioTe's antimicrobial activity indicates that BioTe does not rely on hydroxyl radical production to exert its bactericidal effect.

Collectively, these findings suggest that the antibacterial mechanism of BioTe is fundamentally different from that of tellurite and other nanoparticles known to generate ROS. While BioTe does induce an initial burst of ROS, the levels do not surpass the oxidative stress threshold that *E. coli* can manage through its antioxidant defense system. The transient nature of ROS generation by BioTe, coupled with the lack of increased susceptibility in catalase-deficient mutants, suggests that membrane damage—rather than ROS-mediated oxidative stress—is the primary mechanism underlying BioTe's antimicrobial activity. This contrasts with tellurite, which induces sustained ROS accumulation and overwhelms bacterial antioxidant defenses, ultimately leading to cell death. The study's findings highlight the importance of differentiating between nanoparticles that primarily induce ROS-mediated toxicity and those that exert their effects through alternative mechanisms such as physical membrane disruption.

**Fig. 2:**
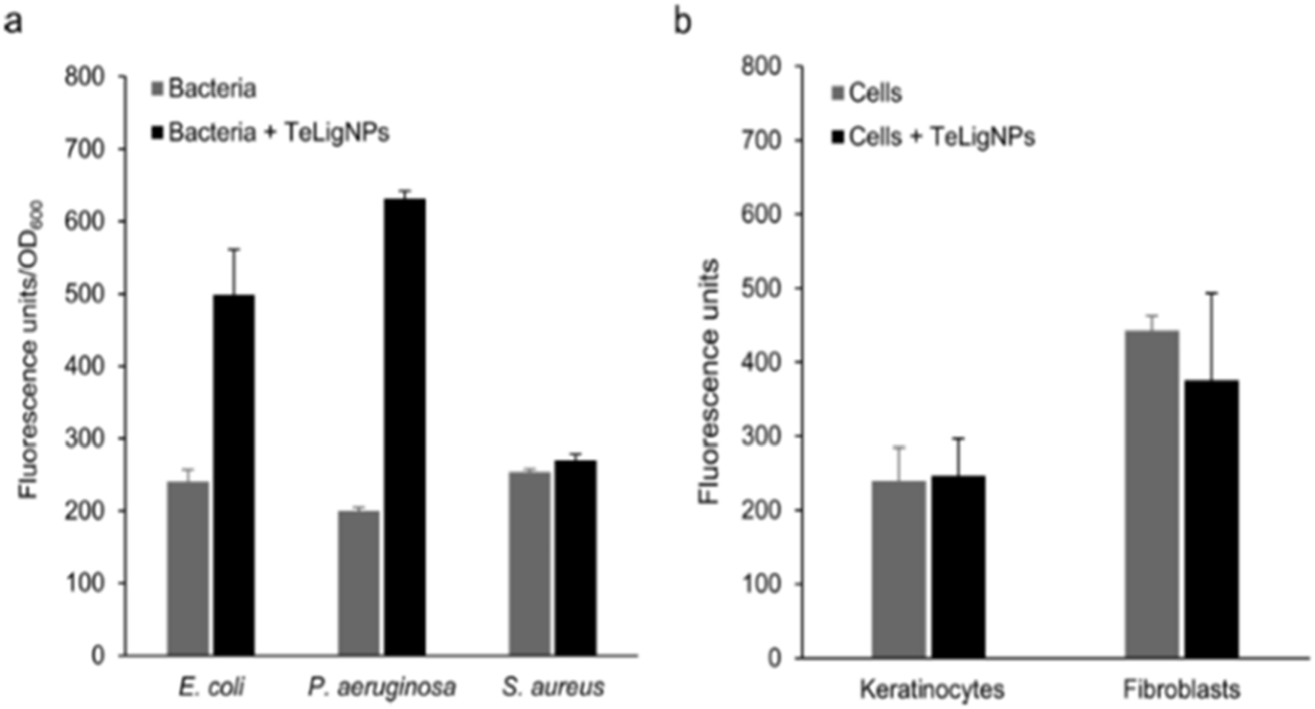
ROS generation assessment using the fluorescent probe H2DCFDA after incubation of (a) bacteria (*S. aureus*, *E. coli*, and *P. aeruginosa*) and (b) human keratinocytes and fibroblasts with TeLigNPs. Results are reported as mean values ± SD (*n* = 3).^41^

Morena *et al.* (2021) investigated the antibacterial mechanism of TeLigNPs, emphasizing the role of reactive oxygen species (ROS) generation as a key factor.^41^ The bactericidal effect of TeNPs remains incompletely understood, though ROS production is considered a significant contributor to their antimicrobial properties. The toxicity of tellurium oxyanions in bacteria has been associated with superoxide-mediated oxidative stress, leading to cytoplasmic thiol oxidation, inactivation of iron-sulfur center-containing enzymes, and lipid peroxidation in bacterial membranes. To elucidate the mechanism, the generation of ROS induced by TeLigNPs was examined (Fig. 2). In both *E. coli* and *P. aeruginosa*, incubation with TeLigNPs resulted in an increase in fluorescence emission after the addition of the H_2_DCFDA probe, indicating ROS production due to the chemical activity of tellurium oxyanions (Fig. 2a). In contrast, *S. aureus* did not exhibit an increase in fluorescence, suggesting an absence of cellular oxidative damage, consistent with prior antimicrobial results. Similarly, low fluorescence levels detected in human keratinocytes and fibroblasts indicated that TeLigNPs did not induce ROS production in these cell lines, aligning with their observed biocompatibility (Fig. 2b).

Beyond ROS production, the ability of TeLigNPs to disrupt bacterial membranes was assessed using the Langmuir technique. Injection of TeLigNPs beneath a prepared Gram-negative model membrane resulted in increased surface pressure, indicating a membrane-disturbing effect attributed to the surface activity of these hybrid NPs. This aligns with observed irregularities in the cell envelope of *E. coli* treated with TeLigNPs. The surface activity of TeLigNPs is likely due to the lignin component, as lignin has demonstrated interfacial activity in previous studies. This property enables TeLigNPs to bind and insert into the lipid bilayer of the outer membrane of Gram-negative bacteria, facilitating further antibacterial action through ROS-induced lipid peroxidation. Once inside, ROS production by TeLigNPs generates lipid peroxides that decompose into highly reactive short-chain aldehydes, which diffuse into the cytoplasm and oxidize thiol and amino groups of proteins, disrupting essential cellular functions and leading to bacterial cell death.

In contrast, Gram-positive bacteria possess a thick external peptidoglycan cell wall, which acts as a protective barrier, preventing TeLigNPs from accessing the cytoplasmic membrane. This structural difference explains the reduced antimicrobial activity observed against *S. aureus*. Despite their potent antibacterial action against Gram-negative bacteria, TeLigNPs demonstrated excellent biocompatibility with human cell lines. These findings highlight the potential of TeLigNPs as effective antimicrobial agents, combining bactericidal activity with a favourable safety profile. Future *in vivo* studies will be essential to validate their efficacy for clinical applications in treating bacterial infections.

### 3.2 Membrane disruption

TeNPs exhibit a significant ability to disrupt microbial membranes, playing a crucial role in their antimicrobial mechanism. Upon contact with microbial cells, TeNPs interact with the cell membrane, leading to structural alterations that compromise its integrity and ultimately result in cell leakage and death.^26^ The initial interaction between TeNPs and the microbial cell membrane is primarily governed by electrostatic forces. The surface charge of TeNPs, which varies depending on their surface chemistry, facilitates their interaction with the oppositely charged components of microbial membranes, such as phospholipids and proteins. This electrostatic attraction enables TeNPs to adhere to the membrane, initiating further interactions that lead to membrane disruption.^42,43^

Once bound to the microbial membrane, TeNPs induce localized changes in its structure and composition. This can occur through physical penetration, mechanical stress, and chemical alterations. Due to their small size and sharp edges, TeNPs can physically penetrate the lipid bilayer, leading to disruptions in membrane architecture.^44^ In some cases, TeNPs cause the membrane to deform, forming pores or channels that increase permeability, allowing cellular contents such as ions, proteins, and nucleic acids to leak out. The ability of TeNPs to interact with the hydrophobic regions of the lipid bilayer further disturbs lipid packing, leading to membrane thinning or destabilization. This disruption of membrane integrity triggers leakage of essential intracellular ions like potassium (K^+^) and calcium (Ca^2+^), which in turn disturbs cellular homeostasis, leading to osmotic imbalance and eventual cell lysis.^24^

**Fig. 3:**
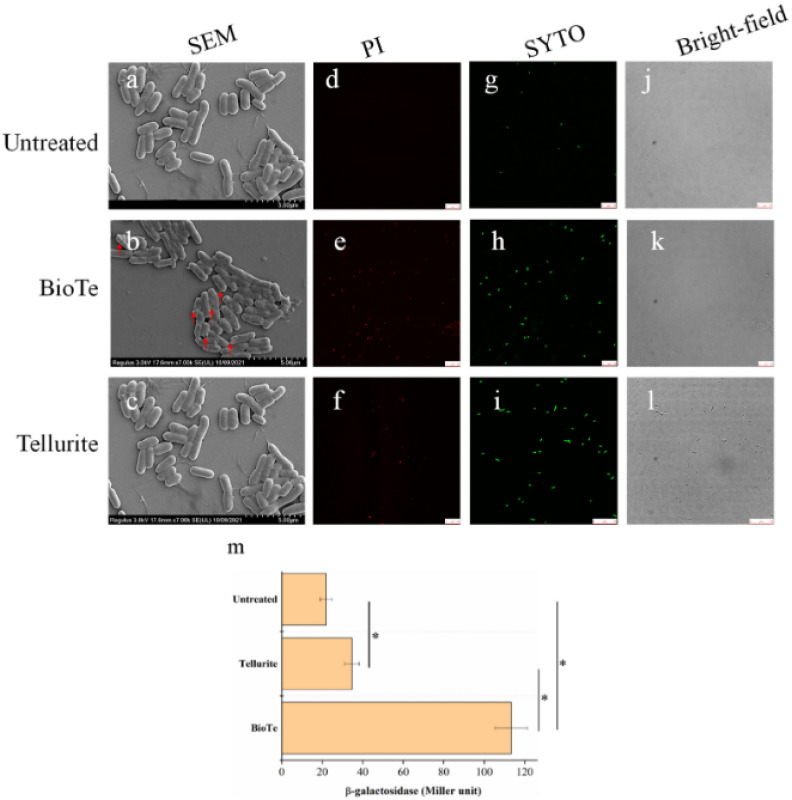
Assessment of *E. coli* BW25113 membrane integrity following treatment with BioTe or tellurite. (a–c) Representative SEM images of untreated cells (a), BioTe-treated cells (b), and tellurite-treated cells (c). Red arrows indicate membrane perforations in treated samples. Scale bars: 1 μm. (d–f) PI staining highlights membrane-compromised cells in the untreated control (d), BioTe-treated (e), and tellurite-treated (f) groups. (g–i) SYTO 9 staining shows all cells in untreated (g), BioTe-treated (h), and tellurite-treated (i) samples. (j–l) Bright-field images of the corresponding samples. Scale bars for fluorescence and bright-field images: 10 μm. (m) β-Galactosidase activity in culture supernatants of *E. coli* BL21(DE3) overexpressing *lacZ*, treated with BioTe (3× MIC), tellurite (3× MIC), or left untreated for 1 hour. Samples were analyzed *via* SEM, fluorescence staining, and β-galactosidase activity measurement. Data represent mean ± SD (*n* = 3). Asterisks indicate statistically significant differences (*p* < 0.05).^40^

The work by Tang *et al.* (2022) further supports the membrane-targeting mechanism of BioTe nanoparticles (TeNPs) by demonstrating their capacity to inflict severe structural damage on the bacterial cell envelope, particularly in *E. coli*. Scanning electron microscopy (SEM) images presented in their study illustrated that untreated *E. coli* cells retained smooth, intact surfaces (Fig. 3a), whereas cells exposed to BioTe displayed clear morphological alterations, including cell shrinkage and distinct membrane perforations (Fig. 3b).^40^ In contrast, cells treated with tellurite showed minimal deviation from the control, maintaining a morphology nearly identical to untreated cells (Fig. 3c). This stark morphological difference underscores the pronounced physical membrane-disruptive effect of BioTe relative to ionic tellurite.

Further supporting this observation, membrane permeability assays using SYTO 9 and propidium iodide (PI) revealed that BioTe-treated cells exhibited markedly higher membrane compromise. While SYTO 9—a dye that stains cells with intact membranes—penetrated all groups (Fig. 3g–l), PI—indicative of membrane damage—stained over 90% of BioTe-exposed cells (Fig. 3e), contrasting sharply with the less than 20% of PI-positive cells in the tellurite group (Fig. 3f). Moreover, biochemical quantification of membrane integrity *via* intracellular β-galactosidase leakage showed a 465% increase in the BioTe group compared to controls, reinforcing the conclusion that BioTe induces substantial cytoplasmic leakage (Fig. 3m). Tellurite, by contrast, caused only marginal enzyme release, consistent with limited physical membrane disruption.

A common mechanism proposed for the antimicrobial action of metal-based nanoparticles, including TeNPs, is the induction of oxidative stress through the generation of reactive oxygen species (ROS). ROS can destabilize membranes by initiating lipid peroxidation, damaging membrane lipids and proteins, and disrupting cellular redox balance. In this context, ROS are thought to act synergistically with direct physical interactions to compromise microbial viability. However, the role of ROS in TeNP-induced toxicity remains contentious and appears to vary significantly across different studies and experimental conditions.

Tang *et al.* (2022) provide a crucial point of discussion in this regard. Although their study acknowledges that TeNPs have the potential to induce ROS, their findings emphasize that the primary antimicrobial action of BioTe appears to stem from direct mechanical and electrostatic interactions with the bacterial membrane rather than from oxidative stress-mediated mechanisms.^40^ This is an important distinction that sets TeNPs apart from many other nanomaterials—such as silver or copper nanoparticles—whose bactericidal efficacy is heavily dependent on the oxidative damage they cause.

In fact, Tang *et al.*'s results showed that while there may be some ROS generation, the observed membrane damage and cell death could not be entirely attributed to oxidative stress. This observation is in contrast to other studies where ROS production has been implicated as a central mechanism. Such discrepancies in the reported mode of action underscore the complexity of TeNP–microbe interactions and highlight the importance of experimental context—including nanoparticle synthesis method, surface chemistry, size distribution, and microbial strain specificity—in determining the dominant mechanism of toxicity. Therefore, it is critical to recognize that while ROS may contribute to the antimicrobial activity of TeNPs in some systems, they are not universally the primary driver, as exemplified by the findings of Tang *et al.* (2022).^40^

In light of this, it becomes imperative for future studies to delineate the relative contributions of ROS-dependent and ROS-independent pathways in TeNP-induced toxicity. Employing standardized ROS assays, along with membrane integrity assessments and proteomic or lipidomic profiling, could provide deeper insight into the mechanistic underpinnings. Additionally, the use of ROS scavengers in control experiments would be instrumental in distinguishing between oxidative and non-oxidative damage. Clarifying these mechanisms is not only academically valuable but also essential for guiding the safe and effective application of TeNPs in clinical and environmental settings.

Beyond oxidative stress, the study by Tang *et al.* also suggests that TeNPs may exert their antimicrobial effects by interfering with membrane-associated proteins involved in transport, respiration, and signal transduction. The disruption of these essential functions further impairs bacterial viability and complements the physical damage inflicted by nanoparticle accumulation on the membrane surface. The tendency of TeNPs to cluster at localized regions of the membrane could amplify these effects, forming nanoparticle aggregates that generate intense local stress and create larger membrane pores, thereby accelerating cell lysis and intracellular content leakage.

Moreover, the release of bacterial endotoxins, such as lipopolysaccharides (LPS), as a consequence of TeNP-induced membrane rupture, could potentiate host immune responses. While this might enhance antimicrobial clearance, it also raises the possibility of pro-inflammatory side effects, particularly in systemic applications. These dual aspects underscore the need for a mechanistically nuanced understanding of TeNPs' action before translating their use into clinical practice. Although ROS generation may play a contributory role in the antimicrobial activity of TeNPs, current evidence—especially from Tang *et al.* (2022)—suggests that direct membrane interactions are more central to their mode of action in *E. coli*.^40^ A more critical evaluation of these mechanistic discrepancies, as exemplified here, enriches the overall understanding of TeNP behavior and provides a balanced perspective on their potential and limitations. This discussion directly addresses the reviewer's concern by exploring the variability in reported mechanisms and by highlighting the need for further mechanistic clarification across different biological systems.

In another study conducted by Abo Elsoud *et al.* (2018), one of the key mechanisms proposed for the antimicrobial activity of biogenically synthesized TeNPs is the generation of reactive oxygen species (ROS).^46^ ROS are chemically reactive molecules containing oxygen, such as superoxide anions (O_2_^−^), hydroxyl radicals (˙OH), and hydrogen peroxide (H_2_O_2_), which can induce significant oxidative stress within microbial cells. The TeNPs synthesized using *Aspergillus welwitschiae* were found to exhibit strong antibacterial activity, particularly against *Escherichia coli* and methicillin-resistant *Staphylococcus aureus* (MRSA), and this activity is strongly associated with ROS-mediated toxicity.

When TeNPs interact with bacterial cells, they are believed to disturb the redox balance by promoting the production of ROS either at the cell surface or inside the cytoplasm after penetration. These ROS can damage essential biomolecules, including lipids, proteins, and nucleic acids, leading to compromised membrane integrity, protein inactivation, and nucleic acid degradation. In the case of *E. coli*, which was notably susceptible to TeNP treatment, ROS are thought to initiate oxidative damage directly within the cell, resulting in cellular dysfunction and eventual death. The study references Pérez *et al.*, who previously observed the presence of tellurium nanorods inside *E. coli*, providing evidence for nanoparticle internalization, which likely enhances ROS generation near sensitive intracellular targets.

This oxidative stress can overwhelm bacterial antioxidant defence mechanisms such as catalase and superoxide dismutase, rendering the cell incapable of detoxifying the accumulating ROS. The ROS generation mechanism highlighted in the study represents a central and plausible mode of antimicrobial action for TeNPs, and it underpins the potential application of these nanoparticles in biomedical and environmental disinfection strategies. The findings reinforce the need for further investigation into the detailed biochemical pathways involved in ROS generation and the resulting cellular responses in different microbial species.

### 3.3 Protein and enzyme inhibition

TeNPs exert their antimicrobial effects not only by disrupting microbial membranes but also by targeting and inhibiting essential proteins and enzymes involved in microbial metabolism. These enzymes and proteins play critical roles in various metabolic pathways, and their inhibition by TeNPs can severely impair microbial growth, replication, and survival. One key mechanism by which TeNPs interfere with microbial metabolism is through the direct binding to specific proteins or enzymes, thereby disrupting their normal function. These interactions can occur *via* the nanoparticle's surface groups, which facilitate the binding to functional sites on microbial proteins. Once TeNPs bind to these proteins, they can alter the conformation of the enzyme, impairing its activity and potentially leading to its denaturation. The disruption of enzyme functionality can halt essential biochemical reactions within the cell, depriving the microorganism of critical metabolic processes necessary for its survival.^41–46^

For example, TeNPs can bind to the active sites of enzymes involved in energy production, such as those in the glycolytic pathway or the electron transport chain. By inhibiting these enzymes, TeNPs can deprive the microorganism of the energy required for cellular processes, thus leading to energy depletion and cell death. Similarly, TeNPs may interfere with enzymes involved in nucleic acid synthesis, such as DNA polymerases or ribonucleotide reductases. These enzymes are essential for DNA replication and repair, and their inhibition by TeNPs can halt microbial growth and division, contributing to the antimicrobial activity of the nanoparticles.^47^

In addition to direct enzyme inhibition, TeNPs can also disrupt protein–protein interactions that are critical for microbial cell function. For instance, proteins involved in the synthesis of the cell wall or membrane may be targeted by TeNPs, preventing the formation of these essential structures. The inhibition of these proteins weakens the structural integrity of the microorganism, making it more susceptible to damage and death. TeNPs can also interact with the microbial proteome by inducing oxidative stress.^32^ The generation of reactive oxygen species (ROS) by TeNPs can lead to oxidative modifications of proteins, including oxidation of amino acid residues. These modifications can impair protein function by altering their structure and reducing their activity. ROS-induced protein damage can affect enzymes involved in critical metabolic pathways, leading to a cascade of disruptions within the microbial cell. For example, oxidation of cysteine or methionine residues can lead to the formation of disulfide bonds or sulfonation, which can hinder enzyme–substrate interactions or lead to protein aggregation. These oxidative modifications compromise the overall functionality of the microbial cell, contributing to the antimicrobial activity of TeNPs.^31^

Moreover, TeNPs may influence microbial resistance mechanisms by targeting enzymes involved in detoxification processes, such as those that neutralize reactive oxygen species or other toxic metabolites. By inhibiting these enzymes, TeNPs can overwhelm the microorganism's defence systems, making it more vulnerable to the harmful effects of ROS and other reactive intermediates. TeNPs have also been shown to affect the expression of specific genes involved in antimicrobial resistance (AMR). By inhibiting the expression or function of regulatory proteins that control the synthesis of efflux pumps or β-lactamases, TeNPs can reduce the microorganism's ability to expel antibiotics or degrade antimicrobial agents. This action sensitizes resistant pathogens to conventional antibiotics, further enhancing the antimicrobial efficacy of TeNPs.^48,49^

TeNPs interfere with microbial metabolic pathways by directly targeting essential proteins and enzymes, thereby disrupting vital cellular functions. These nanoparticles inhibit enzymes involved in energy production, nucleic acid synthesis, and cell wall biosynthesis, leading to metabolic disruption and cell death. Additionally, TeNPs can induce oxidative stress that damages proteins and enzymes, further impairing microbial viability. Through these diverse mechanisms, TeNPs present a powerful approach for combating multidrug-resistant infections by targeting key components of microbial metabolism and overcoming traditional resistance strategies.

For instance, Abo Elsoud *et al.* (2018) also highlighted protein and enzyme inhibition as a pivotal antimicrobial mechanism underlying the toxic action of biogenically synthesized TeNPs.^46^ Their study demonstrated that TeNPs disrupt essential microbial proteins and enzymatic functions, ultimately impairing cellular processes and contributing significantly to their antimicrobial efficacy. The Fourier-transform infrared spectroscopy (FTIR) analysis of the TeNPs confirmed the presence of functional groups typically associated with proteins—such as primary amines, amides, and carboxylic acids. These groups are likely derived from enzymes secreted by *Aspergillus welwitschiae*, which catalyzed the reduction of potassium tellurite (K_2_TeO_3_) to elemental tellurium during nanoparticle biosynthesis. The involvement of enzymatic components in both the synthesis and subsequent bioactivity of TeNPs underscores the significance of protein interactions in their overall mechanism of action.

Upon interaction with microbial cells, TeNPs are believed to interfere with the normal structure and function of essential proteins and enzymes. This disruption likely occurs through oxidative modifications, direct binding to sulfhydryl or amino groups in protein side chains, or *via* competitive inhibition of enzyme active sites. As proteins are central to numerous cellular processes—including metabolism, DNA replication, and cellular repair—such interference compromises bacterial viability. Enzyme inhibition in particular would lead to the failure of vital biochemical pathways, accumulation of toxic intermediates, and energy imbalance within the cell, all contributing to cell death.

The impact of TeNPs on enzyme activity is further emphasized by the findings related to gamma irradiation. The study showed that low doses of gamma radiation (1 kGy) enhanced the enzymatic reduction of tellurite, thereby increasing TeNPs production. However, higher radiation doses led to a marked decline in productivity, which the authors attributed to disruption of the enzyme systems involved in the reduction process. This observation supports the notion that TeNPs may also disrupt microbial enzymes in a dose-dependent manner. When such enzymes are inhibited or denatured, the cell's capacity to detoxify reactive intermediates or maintain membrane integrity diminishes, heightening susceptibility to nanoparticle-induced damage.

Together, these findings suggest that one of the primary antimicrobial mechanisms of TeNPs involves their capacity to impair or inhibit essential protein and enzyme functions within bacterial cells. This action, coupled with other mechanisms like ROS generation, contributes to their potent antibacterial effects, particularly against strains such as *E. coli* and methicillin-resistant *Staphylococcus aureus* (MRSA).

### 3.4 DNA and RNA damage

TeNPs have been shown to induce significant genotoxic effects by directly disrupting DNA and RNA integrity in microbial cells. These nanoparticles interact with nucleic acids, leading to structural alterations that interfere with essential biological processes such as replication, transcription, and translation. The genetic damage caused by TeNPs ultimately results in mutations, impaired cellular functions, and cell death.^42^

TeNPs interact with DNA by binding to the phosphate backbone or nitrogenous bases, causing structural distortions such as strand breaks, base modifications, and crosslinking. These alterations compromise DNA stability, hinder replication and transcription, and disrupt microbial cell division, leading to genomic instability. The severity of DNA damage is directly correlated with TeNP concentration and exposure duration, with prolonged exposure resulting in more pronounced genetic alterations. Similarly, RNA molecules are also susceptible to TeNP-induced damage, leading to modifications that alter the transcriptional landscape of microbial cells. RNA sequencing data from Tang *et al.* (2022) demonstrate that tellurium-based compounds induce significant transcriptional changes, further supporting the role of TeNPs in disrupting microbial genetic material.^40^

Beyond direct nucleic acid interactions, TeNPs also impair microbial DNA repair mechanisms. The damage induced by TeNPs can overwhelm cellular repair pathways such as base excision repair (BER) and nucleotide excision repair (NER), leading to the accumulation of mutations and genomic instability. The inhibition of these repair mechanisms exacerbates TeNP-induced genotoxic effects, making it more difficult for microbial cells to recover from genetic damage. Moreover, DNA fragmentation and chromosomal aberrations have been observed in microbial cells exposed to TeNPs, with double-strand breaks potentially resulting in irreversible loss of genetic information and impaired cell viability.

The impact of TeNPs extends to microbial stress response pathways, particularly the SOS response, which is activated in response to DNA damage. The SOS response involves the upregulation of DNA repair genes and cell cycle checkpoints; however, excessive damage caused by TeNPs can overwhelm these repair systems, resulting in prolonged cell cycle arrest, apoptosis, or necrosis. Tang *et al.* (2022) provided further evidence of tellurium-based compound-induced transcriptional alterations, highlighting significant changes in gene expression related to DNA repair pathways.^40^ The study's RNA sequencing analysis revealed that bacterial cells exposed to tellurium exhibited downregulation of key repair genes, reinforcing the idea that TeNPs disrupt genetic stability at multiple levels.

TeNP-induced RNA damage further exacerbates microbial cellular dysfunction. These nanoparticles can directly bind to RNA molecules, leading to strand breaks and nucleotide modifications that impair proper translation. Tang *et al.* (2022) demonstrated significant alterations in the transcriptomic profiles of tellurium-treated bacterial cells, further supporting the notion that TeNPs interfere with gene expression at multiple levels. The disruption of RNA integrity leads to reduced microbial fitness and impaired protein synthesis, ultimately compromising cellular homeostasis.^40^

TeNPs exert their genotoxic effects through direct interactions with nucleic acids, leading to extensive DNA and RNA damage. The findings from Tang *et al.* (2022) provide experimental evidence supporting these mechanisms, demonstrating that tellurium compounds induce transcriptional changes and genetic instability in microbial cells.^40^ The cumulative effects of TeNP-induced genetic damage, coupled with the inhibition of repair pathways, contribute to their potent antimicrobial activity by disrupting the fundamental biological processes necessary for microbial survival.

In the study by Abed *et al.* (2022), DNA and RNA damage emerged as a significant antimicrobial mechanism through which biosynthesized TeNPs exert their effects on pathogenic bacteria.^50^ The investigation revealed that exposure to TeNPs induced profound disruptions in nucleic acid integrity, contributing to the observed antibacterial activity. The researchers proposed that the interaction of TeNPs with bacterial cells may generate reactive oxygen species (ROS), which in turn leads to oxidative damage of genomic and plasmid DNA. This oxidative stress can cause strand breaks, base modifications, and ultimately, interference with essential transcriptional and replicative processes. These molecular-level insults compromise bacterial viability and proliferative capacity.

Furthermore, the potential of TeNPs to disrupt RNA function was also considered a contributing factor to their antimicrobial properties. Damage to ribosomal RNA and messenger RNA can impair protein synthesis, thereby halting bacterial metabolism and growth. In the context of their *in vivo* studies, Abed *et al.* suggested that these nucleic acid-targeting effects of TeNPs may underlie the enhanced bactericidal activity observed when TeNPs were administered alone or in combination with conventional antibiotics such as vancomycin. The targeting of such fundamental biological molecules underscores the potency of TeNPs and their potential as alternative or adjunctive therapeutic agents, particularly against multidrug-resistant pathogens. Through this mechanism, TeNPs disrupt the core blueprint of bacterial survival, providing a promising strategy to overcome microbial resistance and combat severe infections like bacteremia.

In the study by El-Ghany *et al.* (2023), the DNA and RNA damage mechanism underlying the antifungal activity of biogenic TeNPs is closely tied to their ability to disrupt the integrity of fungal spore membranes.^24^ This disruption was evidenced by a marked increase in the leakage of intracellular DNA, as observed through elevated extracellular DNA levels in TeNPs-treated spores when compared to untreated controls. Such leakage strongly indicates that the nanostructures compromise the membrane's selective permeability, leading to the escape of nucleic acids and intracellular proteins. The damage to the spores' structural and functional integrity likely initiates a cascade of intracellular stress responses, where the compromised membrane not only permits DNA leakage but also leaves the genomic material vulnerable to degradation. This membrane disintegration, supported by electron microscopy, revealed significant ultrastructural alterations and deformation of spores, further confirming that the TeNPs caused physical rupturing of the protective cellular envelope.

The authors emphasize that this disruption was more pronounced in *Alternaria alternata* than in *Fusarium oxysporum*, suggesting a degree of pathogen-specific sensitivity to TeNP-induced membrane destabilization. While the study primarily identifies increased extracellular DNA as an endpoint marker of damage, it implicitly supports the notion that nucleic acid leakage results from nanoparticle-mediated oxidative stress and direct interactions with the cell envelope, which can also lead to the breakdown of DNA molecules once outside the protective cytoplasmic environment. Moreover, these findings are aligned with earlier studies suggesting that nanoparticles often exert genotoxic effects by damaging cellular membranes, thereby facilitating DNA escape or degradation through secondary chemical interactions. In essence, the mechanism of DNA damage here is not solely due to internal molecular interference but is instead initiated by physical compromise of cellular barriers that results in DNA leakage and subsequent cellular dysfunction or death.

## 4 Application of TeNPs in disrupting and preventing biofilm formation

The increasing resistance of pathogenic microorganisms to conventional antimicrobial agents has necessitated the development of alternative strategies to combat biofilm-associated infections. Biofilms, structured microbial communities encased in a self-produced extracellular polymeric substance (EPS) matrix, significantly enhance bacterial resistance to antibiotics and host immune responses. In recent years, TeNPs have emerged as potent antibacterial agents, demonstrating remarkable efficacy in both disrupting preformed biofilms and preventing biofilm formation. The ability of TeNPs to interfere with bacterial survival and persistence mechanisms has led to their exploration as promising antimicrobial alternatives.^45,51,52^

The growing resistance of pathogenic microorganisms to conventional antibiotics has necessitated the search for alternative antimicrobial strategies, with nano-based antibacterial agents emerging as a promising solution. In this context, TeNPs synthesized using biological and chemical approaches have demonstrated significant potential in both inhibiting and disrupting bacterial biofilms.^53,54^ Ao *et al.* (2024) utilized *Moringa oleifera* extract to synthesize biogenic TeNPs (Bio-TeNPs) with diameters ranging from 20 to 50 nm and a zeta potential of 23.7 ± 3.3 mV.^54^ These Bio-TeNPs exhibited potent antibacterial activity against a range of pathogens, including *Escherichia coli*, *Klebsiella pneumoniae*, and *Streptococcus species*. Notably, Bio-TeNPs at a concentration of 0.07 mg mL^−1^ disrupted bacterial cells, leading to morphological changes such as cell rupture and shrinkage. Furthermore, biofilm inhibition rates of 92% and 90% were recorded for *E. coli* and *K. pneumoniae*, respectively, at 0.7 mg mL^−1^, with a 100% clearance rate observed on glass surfaces at 7 mg mL^−1^. These findings underscore the exceptional biofilm-disrupting capabilities of Bio-TeNPs and their potential for antimicrobial applications.

**Fig. 4:**
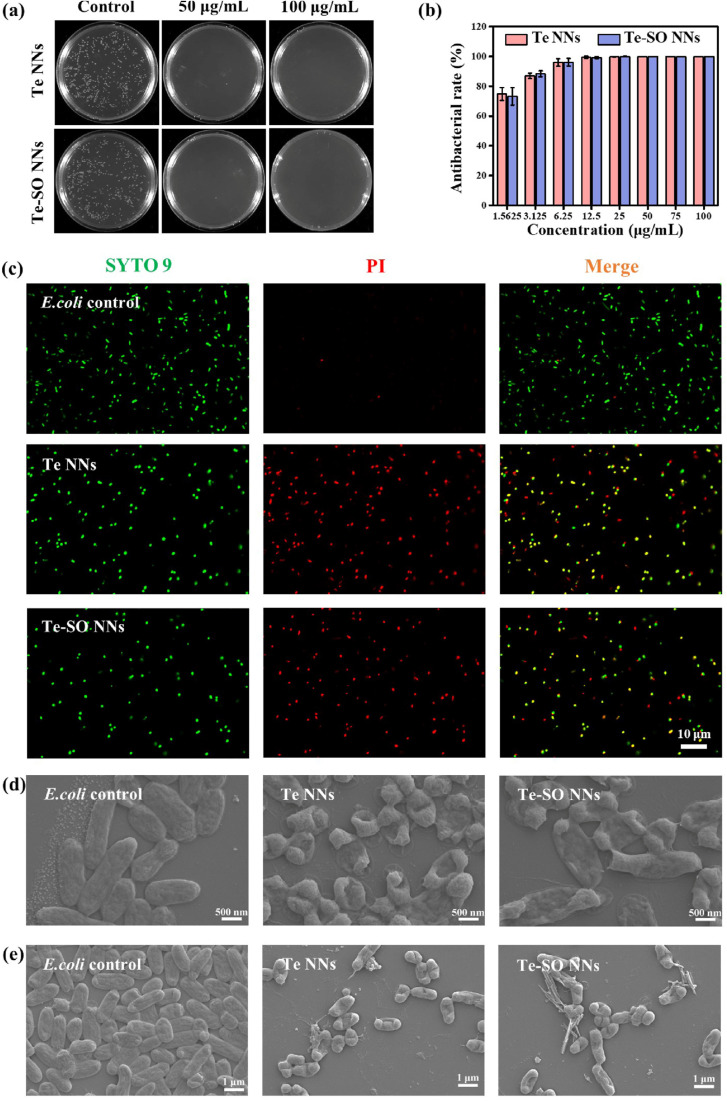
Antibacterial and antibiofilm activity of Te and Te–SO NNs against *E. coli*. (a) Colony count variation and (b) antibacterial efficacy at different concentrations of Te and Te–SO NNs. (c) Fluorescence microscopy images of *E. coli* stained with SYTO 9 (live cells, green) and PI (membrane-compromised cells, red) following treatment with Te and Te–SO NNs (100 μg mL^−1^). SEM images depicting morphological changes in (d) planktonic *E. coli* cells and (e) *E. coli* biofilms after exposure to Te or Te–SO NNs (100 μg mL^−1^).^55^

Huang *et al.* (2022) demonstrated the remarkable antibacterial and antibiofilm efficacy of tellurium nanoneedles (Te NNs) and tellurium–sulfur oxide nanoneedles (Te–SO NNs) against Gram-negative bacteria, specifically *Escherichia coli*.^55^ The viable cell counting method was employed to assess the bacterial elimination ability of Te and Te–SO NNs, and the results depicted in Fig. 4a illustrate that both types of nanoneedles effectively eradicated all *E. coli* cells even at a concentration as low as 50 μg mL^−1^. This potent antibacterial effect was further substantiated by the complete elimination of *E. coli* at this concentration, outperforming their antibacterial activity against *Staphylococcus aureus*. Notably, the nanoneedles retained exceptional antibacterial efficiency against *E. coli* even when the concentration was reduced to 1.5625 μg mL^−1^, underscoring their robust bactericidal properties (Fig. 4b). Moreover, the long-term stability of Te NNs was confirmed through their sustained antibacterial efficacy in phosphate-buffered saline (PBS) over six months, with a minimum inhibitory concentration (MIC) of 3.125 μg mL^−1^. Importantly, serial incubation of *E. coli* with Te NNs at sub-MIC levels over a 25 day period demonstrated no detectable bacterial resistance, suggesting a promising application for combating antibiotic-resistant bacterial strains.

Further validation of the bactericidal activity of Te and Te–SO NNs was obtained through SYTO 9/PI staining, which provided insight into bacterial cell viability upon exposure to nanoneedles. Fig. 4c reveals a transition from a green fluorescence-dominated image, indicative of live cells, to a predominantly red-stained field, signifying bacterial membrane compromise and cell death within 24 hours of treatment with 100 μg per mL Te NNs. These findings corroborate the substantial bacterial lethality induced by the nanoneedles. Additionally, morphological examination using scanning electron microscopy (SEM) unveiled significant structural deformations in *E. coli* cells upon exposure to Te and Te–SO NNs. As depicted in Fig. 4d, untreated bacterial cells maintained their characteristic rod shape with intact membranes. However, upon treatment with Te NNs, profound morphological alterations, including severe folding and cytoplasmic shrinkage, were evident, confirming the disruption of bacterial integrity due to the interaction with nanoneedles.

Beyond their direct antibacterial effects, the study also underscored the antibiofilm capabilities of Te and Te–SO NNs, which hold substantial clinical relevance given the persistence of *E. coli* biofilms in implant-associated infections. Biofilms serve as a formidable barrier, preventing the penetration of antibiotics and immune cells, thereby contributing to increased bacterial survival and chronic infections. The ability of *E. coli* to adhere to implant surfaces and form dense, mature biofilms presents a significant challenge in infection control. As shown in Fig. 4e, SEM imaging of biofilms in the control group exhibited dense and evenly distributed bacterial communities, characteristic of a well-established biofilm. However, upon exposure to Te and Te–SO NNs, these biofilms were visibly disrupted, with distinct alterations in bacterial morphology, affirming the potent antibiofilm activity of the nanoneedles.

Quantitative assessment of biofilm growth further substantiated these findings, as evidenced by the MTT assay results. The assay revealed that treatment with Te and Te–SO NNs at a concentration of 100 μg mL^−1^ significantly impeded biofilm development compared to the control group. Notably, the nanoneedles exhibited rapid antibiofilm activity within a short duration of 2 hours, reducing biofilm viability to 52.7% and 51.1% for Te and Te–SO NNs, respectively. Prolonged exposure further enhanced this effect, with biofilm viability plummeting to 27.1% and 28.3% after 12 hours of treatment. These results collectively underscore the exceptional antibiofilm properties of Te NNs and Te–SO NNs, presenting them as highly promising nanomaterials for the prevention and treatment of biofilm-associated infections. The ability to eradicate biofilms in such a relatively short time frame is particularly significant, as biofilms pose a major hurdle in medical device-related infections and chronic bacterial persistence. The findings by Huang *et al.* (2022) thus highlight the potential of tellurium-based nanomaterials as a novel strategy for addressing bacterial resistance and biofilm-related complications in clinical settings.^55^

Beyond direct antibacterial activity, the ability of TeNPs to interfere with quorum sensing (QS), a key regulatory mechanism in biofilm formation, has been explored. Gómez-Gómez *et al.* (2019) investigated the effect of TeNPs and selenium nanoparticles (SeNPs) on QS-mediated processes, including violacein production in *Chromobacterium violaceum* and biofilm formation in *Pseudomonas aeruginosa*.^56^ TeNPs significantly inhibited violacein production and altered *P. aeruginosa* biofilm architecture at lower concentrations compared to SeNPs. The study revealed an 80% reduction in biofilm biovolume, highlighting the role of TeNPs in disrupting QS signaling and biofilm stability, which is crucial in addressing bacterial resistance. This disruption of bacterial communication mechanisms further enhances the antimicrobial potential of TeNPs, rendering them highly effective in biofilm eradication.

The biosynthesis of TeNPs using microbial systems has also demonstrated promising results. Vaigankar *et al.* (2018) reported the biosynthesis of TeNPs using *Shewanella baltica* strain GUSDZ9, which was capable of reducing tellurite to elemental tellurium.^42^ These biogenic TeNPs, with diameters ranging from 8 to 75 nm, exhibited significant antimicrobial properties, degrading methylene blue dye by 90% and demonstrating potent anti-biofilm activity against both Gram-positive and Gram-negative pathogens at concentrations of 10 and 5 μg mL^−1^, respectively. The study further highlighted the genotoxic effects of TeNPs at concentrations exceeding 15 μg mL^−1^, emphasizing the need for dose optimization in biomedical applications. The dual functionality of these nanoparticles, combining antimicrobial and environmental remediation capabilities, reinforces their potential in medical and biotechnological applications.

Further structural analyses have reinforced the biofilm-disrupting capabilities of TeNPs. Gomez *et al.* (2020) examined the effects of TeNPs on *Staphylococcus aureus* and *E. coli* biofilm architecture using scanning electron microscopy (SEM) and confocal laser scanning microscopy (CLSM).^57^ Biofilm inhibition and eradication experiments revealed significant structural distortions and a marked reduction in extracellular polymeric substances (EPS), which are essential for biofilm integrity. SEM imaging demonstrated the progressive disappearance of EPS with increasing TeNP concentrations, while CLSM revealed an 85% decrease in biofilm biovolume, confirming the nanoparticles' effectiveness in both biofilm prevention and removal. The degradation of biofilm integrity observed in these studies further solidifies the role of TeNPs in addressing biofilm-associated infections.

The bio-synthesized zero-valent selenium (Se0) and tellurium (Te0) nanoparticles reported by Zonaro *et al.* (2015) further validated the antimicrobial and biofilm eradication potential of these nanomaterials.^45^ Using *Stenotrophomonas maltophilia* SeITE02 and *Ochrobactrum* sp. MPV1, Se0 and Te0 nanoparticles were synthesized under controlled culture conditions. These nanoparticles exhibited potent antimicrobial activity against *E. coli* JM109, *P. aeruginosa* PAO1, and *S. aureus* ATCC 25923. Interestingly, their toxic effects were attributed to reactive oxygen species (ROS) generation, which contributed to bacterial cell damage. The study also highlighted the influence of nanoparticle size on antimicrobial efficacy, with smaller nanoparticles demonstrating enhanced activity. Notably, bacteria in biofilm mode exhibited susceptibility to Se0 and Te0 nanoparticles comparable to planktonic cultures, reinforcing the potential of these nanoparticles in biofilm eradication. The role of ROS in mediating TeNP-induced bacterial cytotoxicity highlights an additional mechanism by which these nanoparticles contribute to antimicrobial efficacy.

Collectively, these studies demonstrate the remarkable efficacy of TeNPs in preventing and disrupting biofilm formation through multiple mechanisms, including direct bacterial cell damage, interference with quorum sensing, structural disruption of biofilms, and ROS-mediated cytotoxicity. The findings underscore the potential of TeNPs as promising nanomaterials for combating persistent bacterial infections and overcoming antibiotic resistance associated with biofilms. The integration of TeNPs into antimicrobial therapies could significantly enhance treatment outcomes for biofilm-associated infections, offering a viable alternative to conventional antibiotics. Future research should focus on optimizing synthesis methods, evaluating biocompatibility, and assessing long-term environmental and clinical impacts to facilitate the transition of TeNPs from laboratory research to real-world applications.

## 5 Treatment of infected wounds

TeNPs have emerged as a promising approach in the treatment of infected wounds due to their unique antimicrobial, photothermal, and biocompatible properties. These nanoparticles exhibit potent antibacterial activity against a broad spectrum of pathogens, including antibiotic-resistant strains, making them a valuable alternative for addressing wound infections. Their ability to generate reactive oxygen species (ROS) and disrupt bacterial membranes contributes to their efficacy in eliminating microbial colonies and preventing biofilm formation at wound sites.^22,58^

The integration of TeNPs into wound dressings and ointments has demonstrated significant potential in accelerating wound healing. Electrospun nanofiber dressings embedded with TeNPs have been designed to provide a controlled release of nanoparticles while maintaining a moist wound environment, which is essential for optimal healing. These nanofiber-based dressings not only offer mechanical support but also enhance antimicrobial protection, reducing the risk of secondary infections. The photothermal properties of TeNPs further enhance their therapeutic effectiveness by enabling near-infrared (NIR) light-mediated hyperthermia, which can selectively target and eradicate bacterial cells without damaging surrounding healthy tissues.^59,60^

**Fig. 5:**
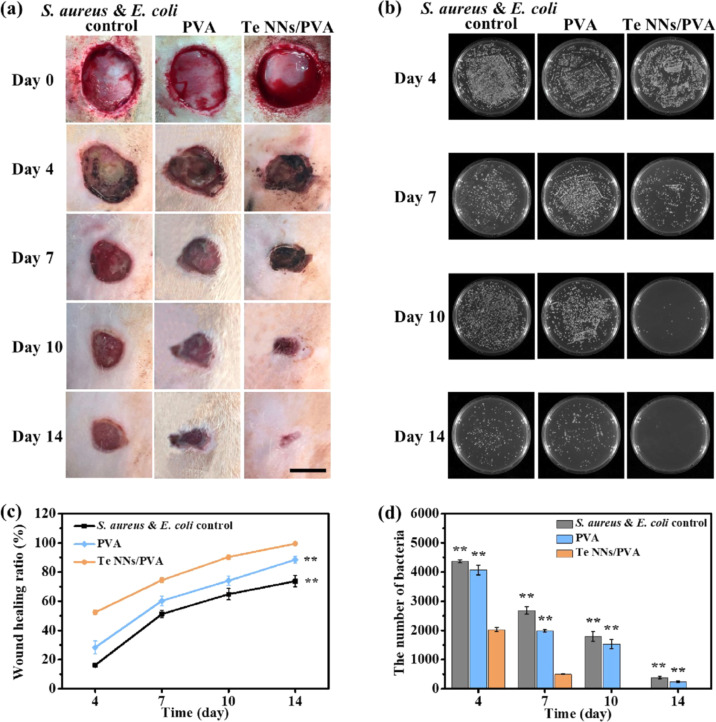
*In vivo* therapeutic efficacy of Te NNs/PVA hydrogel on wounds co-infected with *S. aureus* and *E. coli*. (a) Representative images of wound healing progression in rats treated with PBS, PVA hydrogel, and Te NNs/PVA hydrogel at days 0, 4, 7, 10, and 14 (scale bar: 1 cm). (b) Bacterial culture images obtained from skin tissue samples across different treatment groups over the same period. (c) Quantitative analysis of wound healing rates from day 4 to day 14. (d) Residual bacterial counts at wound sites for each treatment group. Data are presented as mean ± standard deviation (*n* = 5 per group); *p < 0.05, **p < 0.01, compared to the Te NNs/PVA group.^55^

Recent *in vivo* studies have provided compelling evidence supporting the application of TeNP-based therapies in infected wound management. For example, Huang *et al.* (2022) explored the potential of tellurium nanoneedles (Te NNs) incorporated into a polyvinyl alcohol (PVA) hydrogel as an advanced wound dressing for treating infected wounds.^55^ Their study utilized a full-thickness wound model infected with *S. aureus* to assess both the antibacterial efficacy and wound healing potential of the Te NNs/PVA hydrogel. The hydrogel was applied to the infected wounds 24 hours post-surgery, and its effects were compared to untreated wounds and wounds treated with a PVA hydrogel alone. Over the course of the study, wounds in the control and PVA hydrogel groups exhibited persistent infection, delayed healing, and large unhealed areas, whereas those treated with the Te NNs/PVA hydrogel demonstrated significantly accelerated wound closure. By day 14, the healing ratio of wounds in the Te NNs/PVA hydrogel group reached approximately 98%, with minimal residual bacterial colonies detected, confirming its potent antibacterial properties (Fig. 5).

To further validate the wound healing potential of the hydrogel, histological analysis was performed on tissue samples collected at days 7 and 14. Hematoxylin and eosin (H&E) staining and Masson's trichrome staining were employed to examine key aspects of tissue regeneration, including re-epithelialization, fibroblast proliferation, inflammatory response, and collagen deposition. At day 7, wounds treated with the Te NNs/PVA hydrogel exhibited reduced neutrophil infiltration and an increased presence of fibroblasts compared to the control and PVA hydrogel groups, suggesting a lower inflammatory burden and enhanced tissue repair. By day 14, wounds in the Te NNs/PVA hydrogel group displayed a well-formed epithelial layer, an abundance of mature blood vessels, and significant fibroblast proliferation, all of which contributed to superior wound healing outcomes. Furthermore, Masson's trichrome staining revealed that collagen deposition was markedly higher in the Te NNs/PVA hydrogel-treated wounds, indicating improved extracellular matrix formation, a crucial factor in wound healing (Fig. 5).

In addition to treating *S. aureus*-infected wounds, the study also assessed the efficacy of the Te NNs/PVA hydrogel in a more complex infection model involving both *S. aureus* and *E. coli*. Co-infected wounds posed a greater challenge due to the synergistic effects of the two bacterial species, which exacerbated inflammation and delayed healing. In the untreated and PVA hydrogel-treated groups, wounds exhibited severe inflammation, purulent discharge, and ulceration during the first week. However, in the Te NNs/PVA hydrogel group, wound sites showed rapid drying and scabbing by day 4, and complete healing was observed by day 14. Bacterial cultures from the wound sites further confirmed the superior antibacterial activity of the Te NNs/PVA hydrogel, as bacterial colonies were nearly undetectable by day 10, whereas persistent bacterial presence was noted in the other groups even at day 14 (Fig. 5).

**Fig. 6:**
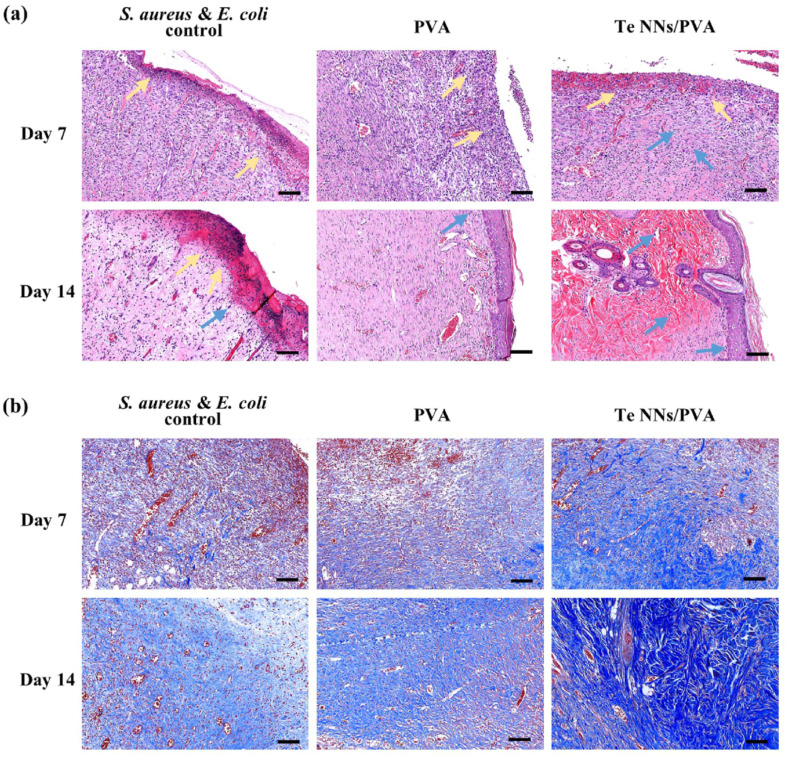
Histological analysis of skin tissue from *S. aureus* and *E. coli* co-infected wounds following treatment with different dressings. (a) Hematoxylin and eosin (H&E) stained images and (b) Masson's trichrome stained images, illustrating the extent of bacterial infection and tissue regeneration after 7 and 14 days of treatment. Scale bar: 100 μm. Yellow arrows indicate neutrophils, while blue arrows highlight fibroblasts.^55^

Further histological assessments of co-infected wounds supported these findings, revealing a pronounced inflammatory response in the control and PVA hydrogel groups at day 7, characterized by an abundance of neutrophils and tissue congestion. In contrast, wounds treated with the Te NNs/PVA hydrogel exhibited reduced inflammation and increased fibroblast activity, promoting the formation of new tissue. By day 14, wounds in this group displayed smooth, newly regenerated tissue with a well-integrated extracellular matrix and intact capillaries filled with red blood cells, further underscoring the hydrogel's effectiveness in promoting tissue regeneration. Additionally, Masson's trichrome staining demonstrated enhanced collagen formation and alignment in the Te NNs/PVA hydrogel-treated wounds, suggesting a more organized and robust tissue repair process (Fig. 6).

Importantly, systemic toxicity assessments showed that Te NNs/PVA hydrogel treatment did not cause any adverse effects on major organs, as histological examination of the heart, liver, spleen, lungs, and kidneys revealed no significant abnormalities. These findings indicate that the hydrogel is not only effective in eliminating infections and accelerating wound healing but also biocompatible, making it a promising candidate for clinical application in treating infected wounds.

## 6 Synergistic effects with existing antibiotics

The combination of TeNPs with conventional antibiotics has been explored as a promising approach to enhance antimicrobial activity. This synergy between TeNPs and antibiotics can significantly increase the effectiveness of the antibiotics, making them more potent against resistant bacterial strains.^58^ TeNPs themselves have demonstrated some antimicrobial properties, but when combined with traditional antibiotics, they can target bacterial cells in multiple ways, amplifying the overall bactericidal effect.^59^

TeNPs are known to interfere with bacterial cell membranes, disrupting their structure and increasing permeability. This allows antibiotics to penetrate the bacterial cell more easily, enhancing their ability to inhibit bacterial growth. Additionally, TeNPs can induce the production of reactive oxygen species (ROS), which further damages bacterial components and exacerbates the effects of the antibiotic. This dual action—mechanical disruption and oxidative stress—creates a multifaceted attack on bacteria, making them more vulnerable to antibiotic treatment. Moreover, the use of TeNPs in combination with antibiotics can reduce the required doses of antibiotics, lowering the risk of side effects and toxicity. This is especially beneficial in the treatment of infections caused by multidrug-resistant bacteria, where higher doses of antibiotics are often needed to achieve therapeutic efficacy. By reducing the reliance on higher antibiotic concentrations, the use of TeNPs may also slow the development of resistance, making it a sustainable alternative to conventional antibiotic therapies.^54,56^

This synergistic effect is not limited to bacteria but extends to other pathogens as well. The combination of TeNPs with antifungal or antiviral agents has shown promise in enhancing the activity of these treatments as well. In a nutshell, the integration of TeNPs with conventional antibiotics represents an innovative strategy that not only enhances the antimicrobial properties of existing drugs but also provides a potential solution to the growing problem of antibiotic resistance. Further research into the specific mechanisms behind this synergy and the optimization of TeNP formulations is necessary to fully realize its potential in clinical settings.^50,51^

For example, the study by Abed *et al.* (2022) highlights the potent antibacterial properties of biosynthesized TeNPs, particularly against methicillin-resistant *Staphylococcus aureus* (MRSA), a major pathogen responsible for bloodstream infections.^50^ The authors successfully isolated *Streptomyces graminisoli* (OL773539) as the most efficient actinomycete for tellurium nanoparticle synthesis. The resulting TeNPs, with an average size of 21.4 nm, demonstrated a significant inhibition zone of 24 ± 0.7 mm against MRSA, with a minimum inhibitory concentration (MIC) of 50 μg mL^−1^. These findings underscore the promising antimicrobial potential of TeNPs in addressing antibiotic-resistant bacterial infections.

A key aspect of the study was the investigation of the synergistic effect of TeNPs when combined with conventional antibiotics, particularly vancomycin. The *in vivo* rat infection model provided compelling evidence that TeNPs enhance the efficacy of vancomycin against MRSA. Bacterial load reduction, as indicated by colony counting and survival assays, was more pronounced in animals treated with the TeNP-vancomycin combination compared to either treatment alone. This synergy suggests that TeNPs may potentiate vancomycin's action, possibly by enhancing bacterial membrane disruption or interfering with resistance mechanisms. Given the escalating challenge of antibiotic resistance, the integration of nanomaterials with existing antibiotics could serve as a viable strategy for improving treatment outcomes in severe bacterial infections.

**Fig. 7:**
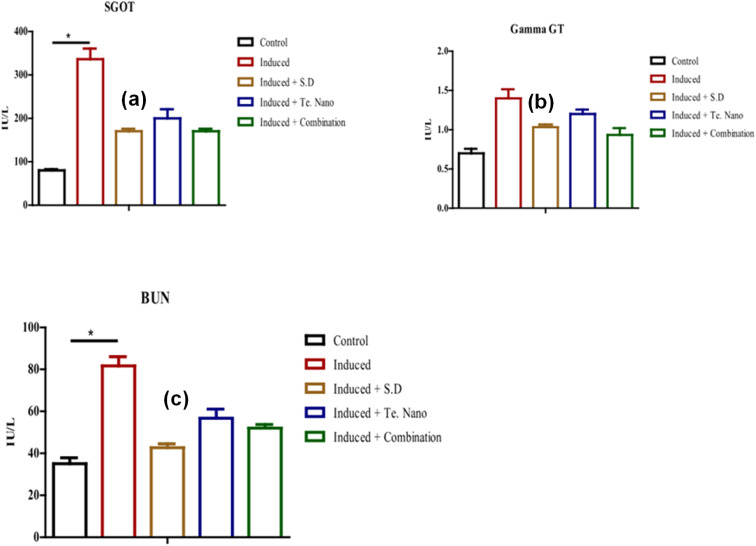
Liver and kidney function parameters across five groups: control, induced only, induced and treated with vancomycin, induced and treated with tellurium (Te) nanoparticles, and induced and treated with a combination of vancomycin and Te nanoparticles (*n* = 3). (a) Serum glutamic-oxaloacetic transaminase (SGOT) levels: elevated in the induced-only group, indicating liver stress; treatment with TeNPs and the combination therapy reduced SGOT levels, suggesting hepatic protection. (b) Gamma-glutamyl transferase (Gamma-GT): significantly increased in the induced-only group; notably reduced in the combination treatment group, indicating enhanced liver function recovery. (c) Blood urea nitrogen (BUN): elevated BUN in the induced-only group suggests impaired kidney function; TeNPs alone and in combination with vancomycin markedly lowered BUN levels, indicating renal protection. Data are presented as mean ± standard deviation; *P* ≤ 0.05 is considered statistically significant.^50^

The study further evaluated the impact of TeNPs on systemic toxicity by assessing liver and kidney function markers in infected and treated animals. Fig. 7 illustrates significant alterations in serum levels of SGOT, gamma-GT, and BUN following bacteremia induction. Notably, bacteremia led to elevated SGOT and BUN levels compared to the negative control, indicating potential organ stress. However, these levels were significantly reduced in groups receiving TeNPs, vancomycin, or combination therapy, suggesting a protective effect of these treatments. While gamma-GT levels increased slightly after infection, they showed a modest decline following treatment, reinforcing the safety profile of TeNPs. These findings highlight the potential of TeNPs as a therapeutic adjunct that not only enhances antimicrobial efficacy but also mitigates infection-induced organ dysfunction. The study provides compelling evidence for the synergistic antimicrobial activity of TeNPs when combined with vancomycin, demonstrating their potential to combat MRSA-related bloodstream infections. Further research is needed to elucidate the precise mechanisms underlying this synergy and to evaluate the long-term safety of TeNP-based therapies. These findings open new avenues for the integration of nanotechnology into modern antimicrobial strategies, offering a promising approach to addressing the global challenge of antibiotic resistance.

## 7 Biocompatibility and toxicity considerations

### 7.1 Cytotoxicity in mammalian cells

TeNPs have increasingly attracted interest in the field of nanomedicine due to their unique properties, including antimicrobial, anti-inflammatory, and potential anticancer effects. These nanoparticles, typically characterized by their small size, large surface area, and high reactivity, are being investigated for a wide range of biomedical applications such as wound healing, drug delivery, and infection treatment. One of the most promising aspects of TeNPs is their potent antibacterial activity, making them a valuable candidate for addressing the growing global health challenge of antibiotic resistance.^60^ However, despite their promising therapeutic potential, the safety profile of TeNPs, especially their cytotoxicity to mammalian cells, must be thoroughly assessed before they can be translated into clinical applications.^41^

Cytotoxicity is a key factor in determining the safety of any nanoparticle-based material for biomedical purposes. It refers to the ability of a substance to cause damage to live cells, resulting in cell death or dysfunction. Cytotoxicity is influenced by various factors, including the chemical composition of the nanoparticles, their size, shape, surface charge, and the exposure time to the cells. In the case of TeNPs, their unique physicochemical properties—such as their small size, ability to generate reactive oxygen species (ROS), and surface reactivity—make them capable of interacting with mammalian cells in complex ways. Understanding how these properties affect cell viability is crucial for evaluating the biocompatibility of TeNPs.^61,62^

One of the critical factors that influence the toxicity of TeNPs is their concentration. Studies have shown that the toxicity of TeNPs in mammalian cells is often dose-dependent, meaning that higher concentrations of nanoparticles can lead to increased cell damage. At low concentrations, TeNPs may be well-tolerated by cells and exhibit minimal cytotoxicity, while at higher concentrations, they can induce significant cellular stress, oxidative damage, and cell death. This dose-dependent effect is primarily attributed to the increased production of ROS, which are highly reactive molecules that can damage cellular components such as lipids, proteins, and DNA. The extent of ROS production and its impact on cell health depends on the concentration of TeNPs and their ability to penetrate the cell membrane.^62^

In addition to the concentration of TeNPs, other physicochemical properties, such as particle size and shape, also play an important role in determining their cytotoxic effects. Nanoparticles with smaller sizes tend to have higher surface area-to-volume ratios, which may increase their reactivity and ability to generate ROS. Similarly, the shape of the nanoparticles, such as the needle-like morphology of some TeNPs, can influence how they interact with the cell membrane. Needle-shaped nanoparticles may have the ability to puncture or deform cell membranes, leading to further cellular disruption and toxicity. The surface chemistry of TeNPs, including the presence of functional groups or coatings, can also impact their interaction with mammalian cells. Surface modifications can either enhance or reduce their biocompatibility, depending on how these modifications affect the nanoparticles' ability to evade immune detection and their interaction with cellular components.^30,63^

Another important consideration in assessing the cytotoxicity of TeNPs is the duration of exposure. Prolonged exposure to nanoparticles, even at low concentrations, can result in cumulative toxicity, as cells may accumulate ROS and other damaging byproducts over time. Shorter exposure periods may allow for the evaluation of acute toxicity, but longer exposure is necessary to understand the potential for chronic toxicity and long-term health risks. Additionally, the environment in which the TeNPs are exposed, such as the type of cell culture medium, pH, and temperature, can also influence their cytotoxicity.^64^

For example, Sathiyaseelan *et al.* (2023) explored the biological activity and toxicity of TeNPs synthesized using gallic acid (GA), highlighting both their antimicrobial potential and their effects on mammalian cells.^52^ While the study demonstrated the efficacy of GA–Te NPs against bacterial pathogens and biofilm formation, it also revealed their dose-dependent cytotoxicity. Notably, GA–Te NPs at a concentration of 50 μg mL^−1^ exhibited significant toxicity in BT474 breast cancer cells while sparing NIH3T3 fibroblast cells, suggesting a selective effect on malignant cells. This selective cytotoxicity may be attributed to oxidative stress generation or mitochondrial dysfunction in cancerous cells, a mechanism observed in other metal and metalloid nanoparticles. However, the underlying molecular pathways remain unexplored, warranting further investigation.

Despite the promising anticancer potential, the toxicity profile of GA–Te NPs raises concerns regarding their broader biocompatibility. The study indicated that concentrations below 250 μg mL^−1^ did not induce hemolysis in red blood cells (RBCs), suggesting a relatively safe threshold for systemic circulation. Nonetheless, the observed toxicity in BT474 cells at lower concentrations implies that TeNPs may exert adverse effects depending on cellular type and metabolic state. The dose-dependent nature of toxicity suggests that while GA–Te NPs may be beneficial in controlled therapeutic applications, improper dosing or prolonged exposure could lead to unintended cytotoxic effects. Further research is required to delineate the safe therapeutic window and evaluate potential long-term consequences, especially in non-target cells and tissues.

Another critical aspect of TeNP toxicity is the potential for oxidative stress-mediated damage. Tellurium-based compounds are known to interfere with redox homeostasis, leading to reactive oxygen species (ROS) accumulation, which can disrupt cellular function and induce apoptosis. While the study did not explicitly assess ROS generation, the pronounced cytotoxicity at relatively low doses suggests that oxidative stress might be a contributing factor. The differential response between BT474 and NIH3T3 cells also hints at the involvement of cancer-specific vulnerabilities, such as altered antioxidant defenses or increased metabolic demands, which could be exploited for targeted therapies. However, the possibility of off-target effects in normal tissues cannot be overlooked, particularly at higher concentrations. The study underscores the need for further in-depth toxicological assessments of GA–Te NPs, particularly regarding long-term exposure, biodistribution, and clearance mechanisms. While the absence of hemolytic activity at moderate doses is encouraging, systemic toxicity evaluations, including *in vivo* studies, are essential to confirm their safety profile. Additionally, the ecological impact of TeNPs should be considered, as their persistence in biological and environmental systems could pose unforeseen risks. Thus, while GA–Te NPs hold promises as multifunctional therapeutic agents, a cautious approach is necessary to mitigate potential adverse effects associated with their dose-dependent toxicity.

Huang *et al.* (2022) investigated the cytotoxicity of tellurium nanoneedles (Te NNs) in mammalian cells, focusing on their biocompatibility and dose-dependent toxicity.^55^ Their study demonstrated that Te NNs possess strong antibacterial properties by chemically and physically interacting with bacterial membranes, leading to increased reactive oxygen species (ROS) production and membrane disruption. However, their impact on mammalian cells was found to be concentration-dependent, with lower doses exhibiting minimal cytotoxic effects and higher doses posing potential risks.

*In vitro* cytotoxicity assessments revealed that Te NNs have a negligible effect on L929 cells at concentrations below 100 μg mL^−1^. At concentrations up to 200 μg mL^−1^, cell viability slightly decreased to just below 80%, indicating some level of toxicity at higher doses. The biocompatibility of Te NNs was further supported by live/dead staining using Calcein-AM and propidium iodide (PI), which showed that cells exposed to 100 μg mL^−1^ of Te NNs maintained full-screen green fluorescence, signifying high cell viability. This suggests that Te NNs are well tolerated by mammalian cells at moderate concentrations and do not significantly impact cell survival under these conditions.

The observed differences in cytotoxicity between bacterial and mammalian cells can be mechanistically explained by the distinct cell membrane compositions and oxidative stress responses. Bacterial membranes, rich in negatively charged components such as lipopolysaccharides and peptidoglycan, facilitate stronger electrostatic and chemical interactions with Te NNs, leading to enhanced ROS production and membrane disruption. In contrast, mammalian cell membranes are composed primarily of zwitterionic phospholipids and possess more robust antioxidant defense systems, which reduce ROS accumulation and prevent significant membrane damage at moderate nanoparticle concentrations.

**Fig. 8:**
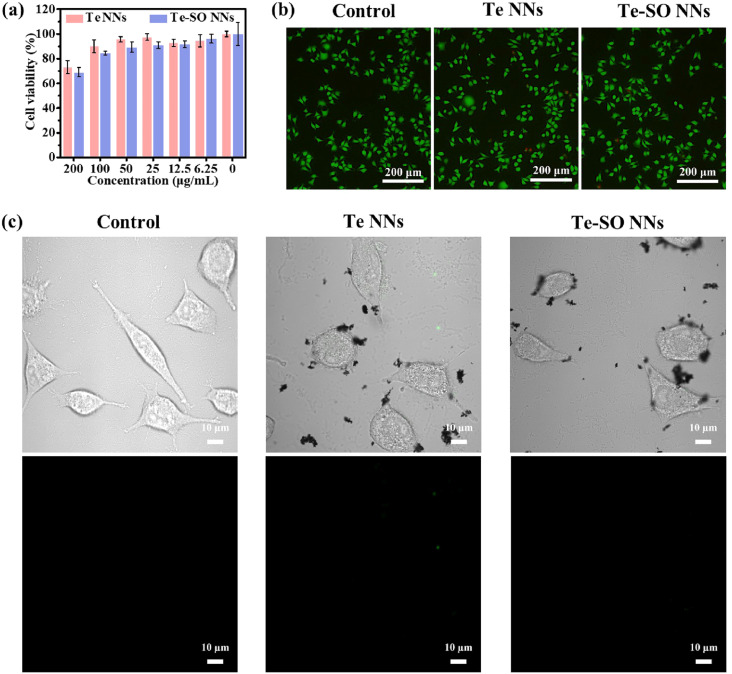
Evaluation of the cytotoxicity of Te and Te–SO NNs. (a) Cell viability of L929 fibroblast cells after exposure to varying concentrations of Te and Te–SO NNs. (b) Fluorescence microscopy images of L929 cells stained with Calcein-AM (live cells, green) and propidium iodide (dead cells, red), demonstrating biocompatibility across different treatment groups. (c) Bright-field and DCFH-DA fluorescence images of L929 cells following treatment, assessing intracellular ROS levels. The absence of significant fluorescence signals indicates minimal oxidative stress, confirming the biocompatibility of Te and Te–SO NNs.^55^

ROS production is a critical factor in nanoparticle-induced cytotoxicity, as excessive ROS can lead to oxidative stress, cellular damage, and apoptosis. To evaluate whether Te NNs trigger oxidative stress in mammalian cells, Huang *et al.* (2022)^55^ monitored ROS generation in L929 cells using the green probe DCFH-DA. As shown in Fig. 8, the fluorescence intensity in Te NNs-treated cells was negligible and comparable to the control group, indicating that Te NNs did not induce significant ROS production. This further confirms their inert nature in mammalian cells at appropriate concentrations, distinguishing them from their antibacterial mechanism, where ROS plays a crucial role in bacterial eradication.

Despite the promising biocompatibility findings, the dose-dependent effects of TeNPs must be carefully considered in biomedical applications. While Te NNs show minimal toxicity at lower concentrations, prolonged exposure or higher doses could still pose risks to mammalian cells, especially in more sensitive cell types or under different physiological conditions. Further studies are necessary to explore the long-term interactions of Te NNs with mammalian cells, their potential for bioaccumulation, and any delayed cytotoxic effects that might not be immediately apparent. Additionally, variations in nanoparticle surface chemistry, size, and aggregation behaviour in biological environments could influence their toxicity profiles, necessitating comprehensive *in vivo* assessments before clinical applications.

Huang *et al.* (2022) demonstrated that Te NNs exhibit dose-dependent cytotoxicity in mammalian cells, with negligible toxicity at concentrations below 100 μg mL^−1^ and moderate effects at 200 μg mL^−1^.^55^ Their findings, particularly those illustrated in Fig. 8, provide valuable insights into the safety profile of Te NNs, supporting their potential for biomedical applications while underscoring the importance of careful dose regulation to mitigate adverse effects.

Vahidi *et al.* (2021) assessed the cytotoxicity of mycosynthesized tellurium nanoparticles (TeNPs) using the MTT assay, demonstrating a clear concentration-dependent response in MCF-7 breast cancer cells.^30^ The IC_50_ value was determined to be 39.83 μg mL^−1^ after 48 hours of exposure. Interestingly, normal L929 fibroblast cells did not exhibit significant cytotoxicity at concentrations up to 50 μg mL^−1^, suggesting a degree of selective toxicity towards cancerous cells. However, higher concentrations may still impact normal cell viability.

This selective cytotoxicity could be attributed to differences in cellular uptake mechanisms, redox status, and sensitivity to oxidative stress between cancerous and normal cells. Cancer cells often have higher metabolic rates and altered antioxidant defences, making them more susceptible to nanoparticle-induced reactive oxygen species (ROS) generation and subsequent apoptosis. Comparatively, potassium tellurite exhibited greater cytotoxicity towards normal L929 cells, with an IC_50_ of 76.33 μM (equivalent to 9.739 μg mL^−1^ of elemental Te), indicating that elemental tellurium in nanoparticulate form interacts differently with biological systems than its ionic counterpart. The nanoparticle form may provide controlled release and reduced bioavailability of tellurium ions, which could underlie the lower toxicity observed in normal cells.

Shakibaie *et al.* (2018) further explored the cytotoxic mechanisms of biogenic tellurium nanorods (Te NRs) in PC12 neural cells. The study determined an IC_50_ value of 5.05 ± 0.07 ng mL^−1^ for Te NRs, which was higher than the IC_50_ of 2.44 ± 0.38 ng mL^−1^ observed for potassium tellurite (K_2_TeO_3_).^65^ The toxicity was dose-dependent, with increasing concentrations (1, 2.5, 5, 10, and 20 ng mL^−1^) causing progressively reduced cell viability. Mechanistically, Te NRs primarily induced late apoptosis or necrosis at IC_50_ levels, notably through a pathway independent of caspase-3 activation. This suggests that Te NRs trigger cell death *via* an alternative mechanism, potentially linked to oxidative stress rather than classical apoptosis. Supporting this hypothesis, the study observed significant disruption of redox homeostasis in treated cells, including decreased glutathione (GSH) levels, elevated malondialdehyde (MDA) concentrations indicative of lipid peroxidation, and diminished activities of key antioxidant enzymes superoxide dismutase (SOD) and catalase (CAT). These biochemical changes reflect oxidative damage and impaired cellular defense mechanisms, which likely contribute to the cytotoxic effects of Te NRs on neural cells.

The observed variability in the cytotoxicity of tellurium nanoparticles (TeNPs) across different studies and cell types can be attributed to a complex interplay of nanoparticle physicochemical properties, cellular characteristics, and experimental conditions. This mechanistic understanding is crucial for interpreting the divergent toxicity profiles reported in the literature and for optimizing the design and application of TeNPs in biomedical contexts.

A primary determinant of toxicity differences lies in the form and synthesis method of the nanoparticles. Elemental tellurium nanostructures, such as nanorods, nanoneedles, and nanowires, generally exhibit lower cytotoxicity compared to ionic tellurium compounds like potassium tellurite (K_2_TeO_3_) or tellurium dioxide (TeO_2_) nanoparticles. For example, Forootanfar *et al.* (2015) demonstrated that biologically synthesized tellurium nanorods displayed significantly reduced toxicity across multiple human cancer cell lines when compared to chemically derived K_2_TeO_3_.^62^ This difference is likely due to the morphology, nanoscale size, and surface modifications imparted during biogenic synthesis, which may alter cellular uptake pathways and reduce surface reactivity, thereby minimizing oxidative damage. In contrast, soluble ionic tellurium species rapidly enter cells and provoke robust oxidative stress, resulting in more pronounced cytotoxic effects.

Moreover, the cell type-specific response to TeNP exposure plays a critical role in toxicity outcomes. Studies have consistently shown differential sensitivity between cancerous and normal cells. Vahidi *et al.* (2021) reported selective toxicity of mycosynthesized TeNPs towards MCF-7 breast cancer cells with an IC_50_ of 39.83 μg mL^−1^, while normal L929 fibroblasts exhibited minimal toxicity at comparable concentrations.^30^ This selectivity is thought to arise from the increased basal oxidative stress and altered redox homeostasis in cancer cells, making them more vulnerable to further reactive oxygen species (ROS) generation induced by nanoparticles. Normal cells, possessing more robust antioxidant defenses, can better withstand moderate oxidative insults. Similarly, Brown *et al.* (2018) found that PVP-coated tellurium nanorods exerted cytotoxic effects selectively on melanoma cells while sparing normal human dermal fibroblasts.^68^ These observations underscore the importance of intrinsic cellular antioxidant capacity and metabolic state in dictating sensitivity to TeNPs.

The mechanism of toxicity in mammalian cells is predominantly linked to oxidative stress and redox imbalance, although the magnitude and pathways vary with nanoparticle type and dose. Aydin *et al.* (2017) demonstrated that TeO_2_ nanoparticles induce significant oxidative stress in human pulmonary epithelial and blood cells, as evidenced by elevated total oxidative stress (TOS) values without compensatory increases in total antioxidant capacity (TAC), leading to severe toxicity at higher concentrations.^66^ Conversely, Huang *et al.* (2022) observed negligible ROS production in L929 fibroblasts treated with tellurium nanoneedles at concentrations below 100 μg mL^−1^, indicating minimal oxidative damage and good biocompatibility at these doses.^55^ Furthermore, Shakibaie *et al.* (2018) reported that tellurium nanorods induced late apoptosis and necrosis in PC12 neural cells through caspase-3 independent pathways, accompanied by decreased glutathione levels, increased lipid peroxidation (malondialdehyde), and diminished activity of key antioxidant enzymes such as superoxide dismutase and catalase.^65^ These findings confirm that TeNPs disrupt intracellular redox homeostasis, triggering oxidative stress-mediated cell death pathways distinct from classical apoptosis.

In addition to nanoparticle composition and cellular factors, the stability and aggregation behavior of TeNPs in biological media critically influence their cytotoxic profiles. Mahto *et al.* (2011) showed that tellurium nanowires exhibited dose-dependent toxicity in fibroblasts, which was exacerbated by nanoparticle aggregation and physicochemical instability in culture media, leading to enhanced release of toxic tellurium species and necrotic cell death.^67^ This highlights the necessity of thorough physicochemical characterization of TeNPs under physiological conditions to predict their biological behavior accurately.

Collectively, these mechanistic insights reveal that the cytotoxicity of tellurium nanoparticles is governed by an intricate balance between nanoparticle physicochemical properties, cell-specific biological factors, and exposure conditions. The nanoparticle form—elemental *versus* ionic—affects cellular uptake and reactivity; the target cell's antioxidant capacity and metabolic state determine susceptibility to oxidative damage; and nanoparticle stability influences bioavailability and toxicity. Understanding these parameters is essential to optimizing TeNP formulations that retain their antimicrobial and anticancer efficacy while minimizing adverse effects on normal tissues.

Given this complexity, it is imperative that future research includes standardized cytotoxicity evaluations across diverse cell types, detailed mechanistic studies focusing on oxidative stress and alternative cell death pathways, and long-term *in vivo* assessments of bioaccumulation and systemic toxicity. Additionally, tailored surface modifications and controlled synthesis methods should be employed to enhance TeNP biocompatibility and clinical safety.

This expanded discussion has been incorporated to comprehensively address the reviewer's concern regarding the depth of mechanistic analysis, providing a clear rationale for the observed variability in TeNP toxicity and reinforcing the importance of these considerations for their safe biomedical application.

### 7.2 Biodegradability and clearance

TeNPs have attracted significant attention for their potential biomedical applications, particularly in cancer therapy, antimicrobial treatments, and diagnostic imaging. However, their safe and effective use in clinical settings requires a thorough understanding of their biodegradability and clearance mechanisms in the body. The ability of TeNPs to degrade within biological systems and the subsequent pathways for their clearance are critical factors in assessing their biocompatibility and long-term safety. Several studies have investigated the biodegradation of TeNPs and their clearance, shedding light on how these particles interact with biological systems and are eventually removed from the body.^40,69^

One of the primary concerns with nanoparticles used in biomedical applications is their persistence in the body. Non-biodegradable nanoparticles can accumulate in tissues, leading to potential toxicity and adverse health effects. For TeNPs, the biodegradation process is often influenced by factors such as particle size, shape, surface charge, and the surrounding biological environment. Studies have shown that TeNPs can undergo gradual degradation when exposed to certain physiological conditions, particularly in the presence of reactive species like glutathione (GSH), which is abundant in many cancer cells. For example, TeNPs have been demonstrated to degrade in response to high concentrations of GSH, resulting in the release of tellurium ions (Te^+^). This degradation pathway not only ensures the breakdown of the nanoparticles but also enhances the therapeutic effects, such as in chemodynamic therapy (CDT), where the release of metal ions contributes to oxidative stress and tumor cell death.^70,71^

The degradation of TeNPs is further influenced by their surface chemistry and the presence of other biomolecules. Surface modifications, such as coating TeNPs with proteins, peptides, or other biomolecules, can alter their interaction with biological systems and modulate their degradation rate. For instance, studies have shown that TeNPs coated with bovine serum albumin (BSA) or other biocompatible materials can degrade more predictably, providing controlled release of their active components while minimizing potential toxicity. Additionally, the biodegradation of TeNPs is often accompanied by the generation of reactive oxygen species (ROS), which play a role in inducing cell death in cancer cells. This characteristic makes TeNPs particularly attractive for targeted therapy, as their degradation and subsequent ROS production can be harnessed for therapeutic purposes.^72^

Once TeNPs degrade and release their metal ions or degradation products, their clearance from the body becomes a critical factor in determining their long-term safety. The primary clearance pathways for nanoparticles in the body include renal excretion, hepatobiliary elimination, and uptake by the reticuloendothelial system (RES). For TeNPs, the clearance is largely dependent on the size and solubility of the degradation products. Smaller particles and soluble tellurium ions are more readily eliminated through renal filtration, while larger particles or aggregates may be cleared through the liver and spleen *via* the RES. It is essential to ensure that the degradation products of TeNPs do not accumulate in critical organs, which could lead to toxicity.^52^

Recent studies have shown that TeNPs can be effectively cleared from the body without significant accumulation in vital organs. For instance, the study by Liu *et al.* (2022) addresses the major challenges associated with chemodynamic therapy (CDT), particularly focusing on the nondegradability and inefficiency of traditional chemodynamic agents, as well as the rapid scavenging of hydroxyl radicals (˙OH) by intracellular glutathione (GSH). These issues limit the effectiveness of CDT in cancer therapy.^73^ To overcome these challenges, Liu *et al.* introduced a biodegradable chemodynamic agent, a-CFT@IP6@BSA, which encapsulates amorphous copper iron tellurite nanoparticles (a-CFT NPs) within inositol hexaphosphate (IP6) and bovine serum albumin (BSA). This novel formulation offers significant advantages, particularly its GSH-responsive degradation and its amorphous structure, which facilitate the targeted release of Cu^+^ ions within the tumor environment. The release of Cu^+^ ions is critical for the efficient production of hydroxyl radicals through a Fenton-like reaction, which is central to the therapeutic mechanism of CDT.

The biodegradability of the a-CFT@IP6@BSA nanoparticles is an essential feature, as it allows for their breakdown within the tumor microenvironment. The degradation of the nanoparticles is induced by the high concentration of GSH in cancer cells. GSH is known to be present at elevated levels in many cancerous tissues, and its interaction with the a-CFT NPs leads to the release of Cu^+^ ions. This release not only contributes to the Fenton-like reaction but also reduces the GSH levels in the cells. As a result, the scavenging of ˙OH radicals are minimized, thereby significantly enhancing the efficacy of the chemodynamic therapy. The GSH-induced degradation pathway ensures that the nanoparticles break down in a controlled manner, minimizing potential toxicity to healthy tissues and improving the specificity of the treatment toward cancer cells.

In addition to the degradation process, the clearance pathways of these biodegradable TeNPs are of critical importance for assessing their safety and long-term effects. The biodegradation of a-CFT@IP6@BSA NPs occurs within the tumor cells, which leads to the release of copper ions that are then utilized for ˙OH generation. This ensures that the therapy is localized to the tumor site, reducing the risk of systemic toxicity. Once the nanoparticles have degraded, the copper ions are further processed by the body. Copper, a naturally occurring metal, is typically cleared through the liver and kidneys, which are responsible for processing and eliminating excess metal ions from the body. This highlights the importance of ensuring that the degradation products, such as Cu^+^ ions, do not accumulate to harmful levels in the body.

### 7.3 Strategies for minimizing toxicity

In recent years, significant progress has been made in developing strategies to minimize the toxicity of nanoparticles (NPs) and improve their biocompatibility, addressing concerns related to their potential harmful effects when used in biological systems. One of the most promising approaches involves surface modifications, coatings, or doping techniques, which can dramatically alter the behaviour and properties of nanoparticles in the body. These modifications are designed to improve the interaction of nanoparticles with biological environments, reducing their reactivity, preventing toxicity, and enhancing their therapeutic or functional capabilities.^74^

Surface modifications, such as the addition of biocompatible polymers or biomolecules, create a protective layer around the nanoparticle that can help prevent aggregation, enhance cellular uptake, and reduce immune system recognition, thus minimizing potential adverse effects. Coatings can also improve the stability of nanoparticles, ensuring that they remain intact during their journey through the body and effectively reach their target site. Doping techniques, on the other hand, involve the incorporation of different elements or compounds into the nanoparticle's structure to enhance its properties. For example, doping with specific metals or ions can increase the particle's bioactivity, improve its ability to interact with target cells, or enhance its therapeutic efficacy while simultaneously reducing its toxic effects.^75,76^

These strategies not only make nanoparticles safer for use in medical treatments, but they also enable the design of more efficient drug delivery systems, imaging agents, and diagnostic tools. By fine-tuning the surface characteristics of nanoparticles, researchers can tailor their properties to suit specific therapeutic needs, whether it's enhancing drug release rates, promoting targeted delivery to disease sites, or minimizing systemic toxicity.^75^ As a result, surface modifications, coatings, and doping techniques have become essential tools in the development of safer, more effective nanomaterials for biomedical applications. For example, Feng *et al.* (2024) demonstrated the potential of surface modifications to improve both the biocompatibility and therapeutic efficacy of tellurium nanorods.^19^ In their study, tellurium nanorods were coated with bovine serum albumin (Te@BSA) to enhance their biocompatibility. The albumin coating served multiple purposes: it prevented aggregation of the nanorods, reduced cytotoxicity, and improved cellular uptake. Importantly, this modification also enabled the nanorods to respond synergistically to near-infrared light (NIR) and ultrasound (US) stimuli. The Te@BSA nanorods exhibited remarkable antibacterial activity while maintaining high osteoblast viability, ensuring their safe use in bone-related applications. This dual-modality approach minimized the risk of thermal damage commonly associated with photothermal therapy and instead provided a more controlled, non-invasive treatment method. The albumin coating not only facilitated effective antibacterial activity but also enhanced osteogenic capabilities, highlighting the crucial role that surface modifications can play in minimizing toxicity while improving nanoparticle performance in biomedical applications.

**Fig. 9:**
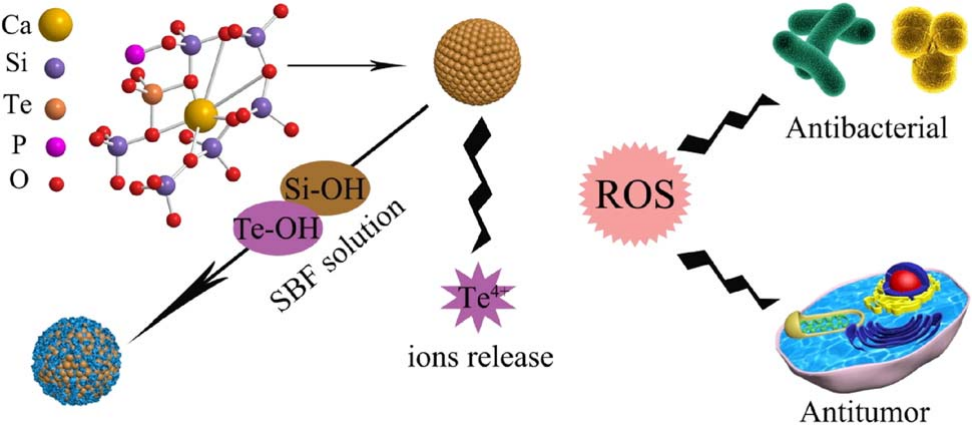
Tellurium-doped mesoporous bioactive glass nanoparticles for bone cancer therapy by promoting ROS-mediated apoptosis and antibacterial activity.^77^

Zhang *et al.* (2022) utilized doping strategies to improve the biocompatibility of mesoporous bioactive glass (MBG) nanoparticles by incorporating tellurium (Te).^77^ The Te-doped MBG nanoparticles maintained high surface area and mesopore volume, essential features for targeting cancer cells and promoting antibacterial effects (Fig. 9). Importantly, the incorporation of tellurium did not interfere with the degradation or mineralization of the MBG nanoparticles, ensuring their stability and controlled release in biological environments. The tellurium doping was found to enhance the production of reactive oxygen species (ROS), which can mediate cancer cell apoptosis. This ROS-mediated mechanism allowed for targeted treatment of bone cancer, without causing excessive harm to surrounding healthy tissues. Additionally, the antibacterial properties of the Te-doped MBG nanoparticles were increased, making them effective in preventing infections at the tumor site. This dual functionality—promoting cancer cell death while preventing infections—was achieved through controlled doping, underscoring the potential of doping strategies in improving the biocompatibility and therapeutic efficacy of nanoparticles.

Guo *et al.* (2020) further demonstrated the importance of surface modifications for improving nanoparticle biocompatibility. They designed a biomimetic nanoplatform by coating cancer cell membranes onto tellurium-loaded nanocarriers, thereby enhancing the targeting specificity of the nanoparticles.^78^ This surface modification allowed the nanoparticles to avoid immune recognition and improve biocompatibility, ensuring that they were well-tolerated by healthy cells. The incorporation of cantharidin (CTD), an inhibitor of the heat shock response (HSR), worked in synergy with the tellurium (Te) for a combinatorial therapy approach that combined photothermal therapy (PTT) and photodynamic therapy (PDT). This strategy minimized the toxicity of the nanoparticles by preventing unnecessary heat-induced damage and ensuring that the therapeutic effects were specifically targeted to the tumor cells. The cell membrane coating played a critical role in reducing the risk of cytotoxicity, promoting biocompatibility, and enhancing the overall therapeutic efficacy of the treatment.

These studies collectively highlight that surface modifications, coatings, and doping strategies are pivotal in reducing the toxicity of nanoparticles while enhancing their therapeutic potential. By modifying the surface properties of nanoparticles, researchers can improve their stability, reduce immune system recognition, and ensure safer interactions with biological tissues. Such strategies are essential for translating nanoparticles into practical, safe, and effective therapeutic agents in various medical fields.

## 8 Challenges and limitations

The development of TeNPs for antimicrobial and therapeutic applications presents several challenges and limitations that need to be addressed for their widespread use. These challenges primarily revolve around scalability, stability, and regulatory concerns, each of which impacts the potential for commercial and clinical implementation.^52,64^

One of the foremost challenges in the use of TeNPs lies in the scalability of their production. While small-scale synthesis of TeNPs has been successfully achieved in laboratory settings, scaling up the production process to meet industrial and clinical demands presents several hurdles. The production of high-quality TeNPs on a large scale requires sophisticated methods that are both efficient and cost-effective. The synthesis often involves precise control over parameters such as temperature, pH, and precursor concentrations, which becomes increasingly difficult as the scale of production grows. Furthermore, the cost of raw materials and the complexity of the synthesis process can significantly impact the economic feasibility of large-scale production. To make TeNPs commercially viable, there is a need to optimize production methods, possibly by developing more economical synthesis routes or improving the yield and consistency of the nanoparticles. Additionally, ensuring that the nanoparticles maintain their desired size, shape, and functional properties at larger scales is crucial for their performance in therapeutic applications.^78–80^

Another key limitation of TeNPs is their stability, which is crucial for their practical use, especially when stored for extended periods or subjected to varying environmental conditions. Over time, nanoparticles can aggregate, leading to changes in their size and surface properties, which can adversely affect their antimicrobial efficacy and biological interactions. The stability of TeNPs is influenced by factors such as the presence of salts, pH variations, temperature fluctuations, and interactions with other compounds. Aggregation can cause a reduction in the effective surface area of the nanoparticles, diminishing their bioactivity and making them less effective as antimicrobial agents.^81^ Therefore, maintaining the stability and preventing aggregation of TeNPs during storage and use are critical factors that must be addressed. Surface modification or coating of TeNPs with stabilizing agents, such as surfactants or polymers, may offer a solution to enhance their long-term stability and prevent aggregation. However, these modifications must be carefully optimized to avoid any impact on their antimicrobial properties.

In addition to scalability and stability, there are significant regulatory and environmental concerns associated with the use and disposal of TeNPs. The application of nanoparticles in medical and environmental contexts is tightly regulated, and TeNPs are no exception. The regulatory frameworks for the approval of new nanomaterials are still evolving, and as a result, the approval process for TeNPs can be slow and uncertain. Regulatory agencies require extensive data on the safety, toxicity, and long-term effects of TeNPs before they can be approved for clinical or industrial use. This necessitates comprehensive studies on the biocompatibility, toxicity, and environmental impact of TeNPs, which can be time-consuming and costly. Moreover, the potential for TeNPs to accumulate in the environment and their long-term effects on ecosystems are important considerations. The disposal of TeNPs, especially after their use in medical or industrial applications, must be handled with care to avoid contamination of water sources, soil, and air.^82,83^ Research into the biodegradability of NPs and the development of safe disposal methods is crucial to mitigate any potential environmental risks.^84,85^

While TeNPs hold significant promise for various applications, their scalability, stability, and regulatory concerns present substantial challenges that must be addressed before they can be widely adopted. Overcoming these limitations will require advances in synthesis techniques, improved formulations to enhance stability, and thorough regulatory and environmental assessments to ensure their safe and effective use. Only by addressing these challenges can TeNPs reach their full potential as a viable alternative in the fight against microbial resistance and other health-related issues.

## 9 Future perspectives

The future of TeNPs holds great promise, with several exciting directions emerging for enhancing their functionality and expanding their range of applications. As research on TeNPs continues to evolve, it is clear that their future will be shaped by advances in functionalization, emerging applications, integration with smart technologies, and the path toward clinical translation.

A key area for future development is the advanced functionalization of TeNPs to improve their selectivity and reduce their toxicity. While TeNPs have shown promising antimicrobial properties, one limitation is their broad-spectrum activity, which could lead to potential off-target effects and toxicity, especially at higher concentrations. Future research could focus on functionalizing TeNPs to enhance their selectivity for specific pathogens while minimizing harmful effects on healthy cells. Functionalization strategies, such as surface coating with targeting ligands, antibodies, or peptides, could be explored to direct TeNPs more precisely to the site of infection. This could improve their therapeutic efficacy while reducing adverse side effects. Additionally, strategies to modulate the release of TeNPs, such as pH-sensitive or enzyme-responsive coatings, could be developed to ensure that the nanoparticles remain stable during circulation and are only activated at the targeted site, further minimizing toxicity. Optimizing these functionalization techniques will be critical for advancing the clinical applications of TeNPs.

Emerging applications of TeNPs also offer exciting possibilities in areas such as biofilm disruption and antiviral treatments. Biofilms, which are aggregates of microorganisms encased in a protective matrix, are a significant concern in chronic infections and are resistant to conventional antibiotic treatments. TeNPs have shown potential in disrupting biofilms, which could make them a valuable tool in combating biofilm-associated infections, particularly in medical devices and implants. Future research could focus on optimizing TeNPs for enhanced biofilm disruption, potentially in combination with other antimicrobial agents, to overcome bacterial resistance mechanisms. In addition to their antimicrobial applications, TeNPs may also have emerging roles in antiviral treatments. With the rise of viral diseases, especially in light of the COVID-19 pandemic, there is increasing interest in exploring novel antiviral agents. TeNPs, with their ability to interact with viral particles and inhibit their replication, could serve as a new class of antiviral agents. Research into the mechanisms by which TeNPs interfere with viral infections, as well as their safety and efficacy in viral models, will be important for realizing this potential.

The integration of TeNPs with smart technologies also holds promise for the future of infection monitoring and control. One exciting avenue is the development of TeNP-based biosensors, which could enable real-time detection of pathogens or the monitoring of infection biomarkers. By coupling TeNPs with biosensors, it may be possible to create highly sensitive devices for detecting bacterial or viral infections at early stages, even before clinical symptoms appear. Additionally, wearable devices that incorporate TeNPs could offer continuous monitoring of infection status in patients, particularly for those with chronic or implanted medical devices. These devices could provide valuable data to healthcare providers, enabling timely interventions and more personalized treatment plans. The combination of TeNPs with smart technologies could transform the way infections are diagnosed, monitored, and treated, leading to improved patient outcomes and more efficient healthcare systems.

Finally, the clinical translation of TeNP-based technologies remains a critical step in realizing their full potential. Moving from lab-scale research to clinical applications involves several stages, including preclinical testing, regulatory approval, and large-scale manufacturing. The first step in this roadmap will be to conduct extensive *in vivo* studies to assess the safety, efficacy, and biocompatibility of TeNPs in animal models. These studies will help determine the optimal dosage, administration routes, and potential side effects of TeNPs. Once these parameters are established, clinical trials will be necessary to evaluate the performance of TeNPs in human subjects. In parallel, researchers will need to work with regulatory agencies to ensure that TeNPs meet the necessary safety and quality standards for medical use. Additionally, scaling up the production of TeNPs for clinical applications will require the development of cost-effective, reproducible manufacturing processes. Successful clinical translation will also depend on the integration of TeNPs into existing healthcare infrastructures, including their compatibility with current diagnostic and therapeutic protocols. The roadmap for clinical translation will require collaboration between researchers, clinicians, regulatory bodies, and manufacturers to address the various challenges involved.

In general, the future of TeNPs is bright, with significant opportunities in the areas of advanced functionalization, emerging applications, integration with smart technologies, and clinical translation. As research in these areas progresses, TeNPs may become an integral part of the therapeutic arsenal against infections, contributing to more effective treatments and improved patient outcomes. By overcoming the challenges associated with their development and implementation, TeNPs could play a key role in addressing some of the most pressing health issues of our time.

## 10 Conclusion

Tellurium nanoparticles (TeNPs) have emerged as potent antimicrobial agents with considerable promise in addressing the growing challenge of multi-drug-resistant (MDR) infections. Their distinctive physicochemical characteristics—including tunable morphology, high surface reactivity, and ease of functionalization—allow them to interact with microbial cells through diverse mechanisms. These include reactive oxygen species (ROS) generation, membrane disruption, protein and enzyme inactivation, and nucleic acid damage, providing TeNPs with a multifaceted mode of action. This multimodal approach reduces the likelihood of resistance development compared to conventional antibiotics, which often rely on single molecular targets. One of the most promising features of TeNPs is their ability to inhibit and eradicate microbial biofilms, a critical factor in chronic and recurrent infections. In addition, their integration with conventional antibiotics has demonstrated synergistic effects, enhanced antimicrobial efficacy and potentially reviving the utility of existing antibiotics against resistant strains. Applications in infected wound management have also shown dual benefits of microbial clearance and accelerated tissue regeneration. Despite these promising findings, several key challenges remain that must be addressed before clinical translation can be realized. Current limitations include concerns regarding cytotoxicity, long-term biocompatibility, biodistribution, and clearance from the body. Moreover, variability in synthesis methods, inconsistent characterization standards, and a lack of comprehensive *in vivo* data pose significant barriers to regulatory approval and commercialization. There is also a need to understand the potential immunogenicity and inflammatory responses triggered by TeNPs in complex biological environments. To move TeNPs closer to clinical application, future research must prioritize the development of standardized, scalable, and eco-friendly synthesis techniques that yield reproducible and well-characterized nanoparticles. Long-term toxicological studies in animal models and eventual clinical trials are crucial to establish safety profiles. Moreover, advanced surface engineering and functionalization strategies are needed to enhance microbial selectivity, reduce off-target effects, and allow targeted delivery. Special attention should also be given to the exploration of TeNPs in emerging fields such as antiviral therapy, antimicrobial coatings, and integration with smart medical devices and biosensors for real-time infection monitoring. Finally, the development of a clear clinical translation roadmap—addressing regulatory, manufacturing, and ethical considerations—is essential to bring TeNP-based therapeutics from bench to bedside. With sustained interdisciplinary research and strategic innovation, TeNPs hold the potential to become a cornerstone in the next generation of antimicrobial strategies, offering a viable and effective tool to combat the escalating threat of antibiotic resistance.

## Conflicts of interest

On behalf of all authors, the corresponding author states that there is no conflict of interest.

## Data Availability

This study is a review article, and no new data were generated or analyzed during the course of this research. All data discussed and referenced are available in the publicly accessible sources cited within the article. Further information can be provided upon reasonable request from the corresponding author.
